# Pharmacological activation of TAZ enhances osteogenic differentiation and bone formation of adipose-derived stem cells

**DOI:** 10.1186/s13287-018-0799-z

**Published:** 2018-03-07

**Authors:** Yumin Zhu, Yaping Wu, Jie Cheng, Qiong Wang, Zhongwu Li, Yanling Wang, Dongmiao Wang, Hua Wang, Weibing Zhang, Jinhai Ye, Hongbing Jiang, Lin Wang

**Affiliations:** 1 Jiangsu Key Laboratory of Oral Disease, Nanjing Medical University, Jiangsu, 210029 China; 2 Department of Oral and Maxillofacial Surgery, School of Stomatology, Nanjing Medical University, Jiangsu, 210029 China; 3 Department of Oral Orthodontics, School of Stomatology, Nanjing Medical University, Jiangsu, 210029 China

**Keywords:** Adipose-derived stem cells (ADSCs), Hippo, TAZ, Osteogenesis, Bone regeneration

## Abstract

**Background:**

Adipose-derived stem cells (ADSCs) are an attractive cell source for bone tissue engineering and have great potential for bone regeneration and defect repair. The transcriptional coactivator with PDZ-binding motif (TAZ) has been demonstrated to modulate osteogenic and adipogenic differentiation of mesenchymal stem cells. However, its roles during ADSC differentiation and therapeutic potentials for bone regeneration have as yet not been well established.

**Methods:**

TAZ expression was measured during osteogenic differentiation of ADSCs in vitro. Both loss-of-function and gain-of-function approaches by TAZ knockdown or enforced overexpression were utilized to determine its functions during osteogenic differentiation of ADSCs. TM-25659, a chemical activator of TAZ, was used to determine whether pharmacological activation of TAZ in ADSCs enhanced osteogenic differentiation in vitro and bone formation in animal models. The molecular mechanisms underlying TAZ in promoting osteogenesis of ADSCs were also explored.

**Results:**

Increased TAZ expression was observed during osteogenic differentiation of human ADSCs. TAZ knockdown resulted in compromised osteogenic differentiation and enhanced adipogenic differentiation of ADSCs. In contrast, enforced TAZ overexpression yielded increased osteogenic differentiation and bone regeneration in vivo, and impaired adipogenic differentiation of ADSCs. Pharmacological activation of TAZ by its chemical activator TM-25659 facilitated osteogenic differentiation of ADSCs. Noticeably, transient treatment of ADSCs with TM-25659 or intraperitoneal injection of TM-25659 significantly enhanced bone regeneration of ADSCs loaded with porous β-TCP in vivo. Mechanistically, TM-25659 exposure significantly promoted TAZ phosphorylation and nuclear translocation, and potentiated the assembly of the TAZ-Runx2 complex. Subsequently, the TAZ-Runx2 complex was further recruited to the promoter of osteocalcin and in turn enhanced its transcription.

**Conclusions:**

Our findings indicate that TAZ is a key mediator that promotes ADSC commitment to the osteoblast lineage. Pharmacological activation of TAZ in ADSCs might become a feasible and promising approach to enhance bone regeneration and repair.

**Electronic supplementary material:**

The online version of this article (10.1186/s13287-018-0799-z) contains supplementary material, which is available to authorized users.

## Background

Bone defects due to congenital abnormalities, trauma, or ablative cancer surgery are associated with major functional, esthetic, and psychological problems. Regenerative bone repair with a tissue-engineering approach based on multipotent stem cells has revolutionized current treatment modalities and has been increasingly recognized as a promising therapeutic option for bone repair and regeneration [1]. Bone tissues grown from culture-expanded stem/progenitor cells via in vivo transplantation with scaffolds have been successfully demonstrated to have great translational significance [2, 3]. However, how to fully exploit the therapeutic potentials of stem cells for bone repair and regeneration still represents an unmet challenge and is at the forefront of regenerative medicine.

Adipose-derived stem cells (ADSCs) are multipotent progenitor cells with self-renewal capabilities and multilineage differentiation potentials including osteogenesis, adipogenesis, and chondrogenesis [4]. Mounting evidence has established that ADSCs hold significant promise for regenerative therapies largely due to their convenient isolation, abundant sources, lack of immunogenicity, and minor donor morbidity [5, 6]. To achieve their therapeutic potential and utility depends on the understanding of the molecular mechanisms driving their differentiation [7]. It has been increasingly appreciated that cell fate decision of ADSCs relies on a tightly orchestrated activation of lineage-specific genes and repression of genes associated with stemness or other lineages [8]. Among the modulators for ADSC differentiation, these DNA-binding transcriptional factors play essential roles in controlling lineage commitment. For example, runt-related transcription factor 2 (Runx2) and Osterix mediate ADSCs into osteoblasts, while peroxisome proliferator-activated receptor-γ (PPARγ) drives ADSCs to adipocytes [8, 9]. To harness the therapeutic potential of ADSCs for bone regeneration, several biomedical approaches have been proposed to enhance the osteogenic capacity of ADSCs, such as genetic modifications of stem cells, usage of osteoinductive scaffold, and selective chemical compounds, among others [10–13]. Of great interest, stem cell differentiation biased by a pharmacological or chemical approach using small molecules appears to a novel and favorable way to promote regeneration with superior translational potentials which might circumvent limitations of genetic manipulation and ethical concerns [12, 14].

Transcriptional coactivator with the PDZ-binding motif (TAZ, also known as WWTR1) is one downstream effector of Hippo signaling and is involved in cell proliferation, stem cell self-renewal and differentiation, and tumorigenesis [15]. Upon Hippo pathway activation, TAZ and its paralog, YAP (Yes-associate protein), were phosphorylated by a kinase cascade consisting of MST1/2 and LATS1/2. Subsequently, they were retained in cytoplasm and in turn degraded via β-TrCP-mediated proteasomal degradation. When the Hippo pathway is switched off, TAZ/YAP are less phosphorylated and translocated into the nucleus, and form functional transcriptional complexes with various transcriptional factors such as the TEAD (TEA-ATTS DNA-binding domain 1-4) family, Runx2, PPARγ, Smads, and p73 to regulate transcription of downstream targets [15, 16]. Noticeably, TAZ has been identified to be required for mesenchymal lineage commitment by interacting with Runx2 or PPARγ to drive osteoblast differentiation while coordinately repressing adipogenic differentiation [17, 18]. The canonical Wnt signaling promotes mesenchymal stem cell (MSC) differentiation into osteoblasts and inhibits adipogenic differentiation through TAZ stabilization and upregulation [19]. Moreover, zinc-finger transcriptional factors Snail/Slug form complexes with TAZ/YAP and in turn activate TEAD or Runx2 downstream targets to maintain homeostasis and drive osteogenesis of the skeletal stem cell [20]. Recently, an amplification feedback loop between ABL1 (Abelson murine leukemia via oncogene 1) and TAZ has been identified to be required for osteoblastogenesis and embryonic skeletal formation [21]. Transgenic mice that overexpress TAZ in osteoblasts significantly increased bone formation in vivo by enhancing Runx2 and transforming growth factor (TGF)-β signaling [22]. These abovementioned findings highlight the importance of TAZ during osteogenic differentiation of stem cells from a diverse source, and which might be exploited to enhance the capacity of stem cells for bone regeneration. Indeed, several growth factors, cytokines, and chemical or bioactive compounds have been found to stimulate osteoblast differentiation through TAZ-mediated transcriptional activation in a broad spectrum of cell types [23–26].

Here, we sought to determine the roles of TAZ during osteogenic differentiation of human ADSCs and whether pharmacological activation of TAZ in ADSCs can promote osteogenesis and bone regeneration in vitro and in vivo.

## Methods

### Human ADSC harvest and cell culture

All protocols involving human subjects were reviewed and approved by the Institutional Review Board of Nanjing Medical University (2016-0156). Human ADSCs were harvested from lipoaspirate in four healthy donors (two males and two females, mean age 26.6 years) who received abdominal liposuction for esthetic purposes as described previously [13, 27]. Written informed consent was obtained from each patient and information regarding patient age, sex, and health conditions was also recorded. Briefly, specimens were washed in phosphate-buffered saline (PBS) and digested with a 0.075% type II collagenase (Sigma) in Hank’s solution at 37 °C under agitation for 30–60 min. After the collagenase was inactivated by10% fetal bovine serum (FBS; Gibco), the stromal vascular fraction (SVF) was then pelleted by centrifuge. Then, the cell pellet was resuspended, filtered through a 100-μm strainer (BD Biosciences), and seeded in culture dishes. Primary cultures were established and maintained in Dulbecco’s modified Eagle’s medium (DMEM) supplemented with 10% FBS and 1% antibiotics in a humidified incubator with 5% CO_2_ at 37 °C. These ADSCs in passage 3 were subjected to fluorescence-activated cell sorting (FACS) to determine their identity and purity via cell surface marker profiling. ADSCs with different numbers in early passages (3–4 passage) were used for both in vitro and in vivo experiments. Most experimental data were derived from experiments using ADSCs from three independent donors, unless stated otherwise. Human bone mesenchymal stem cells (BMSCs) were purchased from Cyagen Biosciences Inc. (HUXMA-01001, China) and maintained following the manufacturer’s instructions. HEK293T cells were obtained from ATCC.

### Reagents, antibodies, and vectors

The TAZ chemical activator TM-25659 was kindly provided by Prof. Jin Hee Ahn at the Korea Research Institute of Chemical Technology [24, 28]. TM-25659 was dissolved in dimethyl sulfoxide (DMSO) as a stocking solution and diluted with culture medium before adding to cells. All chemicals were purchased from Sigma-Aldrich, unless stated otherwise. The following antibodies were used: TAZ (BD Biosciences, 560,235; Cell Signaling Technology, #8418), Phospho-TAZ (Ser89) (Cell Signaling Technology, #75,275), YAP (Cell Signaling Technology, #14074), Phospho-YAP (ser127) (Cell Signaling Technology, #13008), Runx2 (Santa Cruz Biotechnology, sc-10,758), osteopontin (OPN; Santa Cruz Biotechnology, sc-21,742), osteocalcin (OCN; R&D Systems, MAB1419), glyceraldehyde-3-phosphate dehydrogenase (GAPDH; Santa Cruz Biotechnology, sc-25,778), and H3 (Abcam, ab1791).

The lentiviral vector containing short hairpin (sh)RNAs targeting human TAZ was prepared and the sequence and knockdown efficiency was verified as described previously [29]. The target sequences for shRNA were 5’-AGGTACTTCCTCAATCACA-3′. The lentiviral vector containing human TAZ cDNA was engineered and verified as described previously [29]. Lentiviral particles were prepared by co-transfecting HEK293T cells with lentiviral constructs together with packaging and envelope plasmids (pCMV-VSV-G and pCMV-Δ8.2) coupled with subsequent filtration and concentration. The stable cell clones with TAZ knockdown or overexpression were selected by the corresponding antibiotics and the survivals were pooled for further studies.

### Osteogenic and adipogenic differentiation of ADSCs in vitro

The osteogenic and adipogenic differentiation of ADSCs was performed as previously reported [13, 27]. In brief, for osteogenic differentiation of ADSCs, cells were induced with osteogenic medium containing 50 μM ascorbic acid, 10 nM dexamethasone, 10 mM β-glycerophosphate, and 2% FBS in low-glucose DMEM. At days 7, 14, and 21 after induction the cells were harvested for osteogenic assessments. For adipogenic differentiation of ADSCs, cells were cultured with adipogenic medium containing 1 μM dexamethasone, 50 μM indomethacin, 500 nM IBMX, 5 μg/ml insulin, and 2% FBS in low-glucose DMEM. At day 14 after induction the cells were harvested for adipogenic assessments.

### Cell proliferation and apoptosis assays

Cell proliferation or viability was monitored by absorbance using the MTT assay, while cell apoptosis was determined by flow cytometry using the Annexin V-FITC Apoptosis Detection Kit (Invitrogen) as described previously [13, 30].

### Alkaline phosphatase, Alizarin Red, von Kossa, and Oil Red staining

Follow the induced osteogenic differentiation of ADSCs in vitro at day 7, alkaline phosphatase (ALP) activity was determined using an ALP Staining Kit (Beyotime, Shanghai, China). ALP activity relative to the control was calculated after normalization to the total protein content. For detecting mineralization after osteogenic differentiation at the indicated time points, cells were fixed with 70% (vol/vol) ethyl alcohol (ETOH) and then stained with 2% (vol/vol) Alizarin Red (Sigma-Aldrich). To quantitatively measure calcium deposition, Alizarin Red was destained with 10% (vol/vol) cetylpyridinium chloride in 10 mM sodium phosphate for 30 min at room temperature. The concentration was determined by absorbance measurement at 562 nm on a multiplate reader using a standard calcium curve in the same solution. The final calcium abundance in each well was further normalized with the total protein concentration. For von Kossa staining, cells were cultured in osteogenic medium for 28 days, fixed with 4% paraformaldehyde and incubated with 5% silver nitrate solution for 30 min in the dark, followed by exposure to UV light. For Oil Red staining, cells were cultured in adipogenic medium for 14 days. Oil Red O staining was performed to detect the lipid droplets using the Lipid (Oil Red O) Staining Kit (Sigma-Aldrich) as per the manufacturer’s protocol.

### RNA extraction and quantitative real-time polymerase chain reaction analysis

Total RNA was isolated from cells with Trizol reagents (Invitrogen) and then subjected to reverse transcription and polymerase chain reaction (PCR) using the PrimeScript™ RT-PCR kit according to the manufacturer’s protocol (Takara). The gene-specific primers for human TAZ, Runx2, ALP, OPN, OCN, and GAPDH were listed in Additional file 1 (Table S1). Relative mRNA expression was quantified compared with GAPDH using the comparative CT method.

### Western blot analysis and immunoprecipitation assay

Cells were lysed with ice-cold radioimmunoprecipitation assay (RIPA) buffer containing protease inhibitor cocktail (Roche). Protein lysates were resolved by sodium dodecyl sulfate-polyacrylamide gel electrophoresis (SDS-PAGE), and then transferred onto polyvinylidene difluoride (PVDF) membranes (Millipore). After 5% nonfat dry milk or bovine serum albumin (BSA) blocking and overnight incubation with primary antibodies, these blots were detected using appropriate secondary horseradish peroxidase-conjugated secondary antibodies (Invitrogen) and visualized by an enhanced chemiluminescence detection kit (Pierce). The protein-protein interaction was determined by protein immunoprecipitation using the Pierce™ Co-Immunoprecipitation Kit (ThermoFisher) performed according to the manufacturer’s protocol.

### Chromatin immunoprecipitation (ChIP) assay

The ChIP assay was performed using the Simple ChIP Assay Kit (Cell Signaling Technology) according to the manufacturer’s instructions. Briefly, cells were cross-linked with 1% formaldehyde, harvested and lysed, and then sonicated to shear DNA. The DNA-protein complexes were then isolated with antibodies against TAZ (Cell signaling Technology, #4883) or isotype IgG (Santa Cruz Biotechnology). The protein/DNA complexes were then eluted and reverse cross-linked. Spin columns were used to purify the DNA. The precipitated DNA was quantified by quantitative RT-PCR. Relative enrichment was calculated as the amount of amplified DNA normalized to input and relative to values obtained after normal IgG immunoprecipitation. Primer pairs used for human OCN promoter were as follows: forward 5’-AAATAGCCCTGGCAGATTCC-3′ and reverse 5’-CAGCCTCCAGCACTGTTTAT-3′.

### Luciferase assay

To generate the OCN promoter reporter, the promoter sequence (1252 bp) upstream of the transcriptional start site of human BGLAP (encoding OCN protein) was cloned into luciferase reporter plasmid and verified with direct sequence (pGL-OCN). The 293 T cells (2 × 10^5^ cells/well in 24-well plates) were transiently transfected with pGL-OCN, TAZ, constitutively active TAZ (TAZ^S89A^), or TEAD4 plasmids and phRL-CMV plasmid (Promega) using lipofectamine 3000. Cells were lysed 24 h after transfection and assayed for firefly and *Renilla* luciferase activity using the Dual-Luciferase reporter system (Promega). The data are expressed as the ratios of firefly to *Renilla* activity.

### In vivo osteogenesis of ADSCs and heterotopic bone formation

Two million ADSCs with stable TAZ overexpression were initiated towards osteogenic differentiation by osteogenic medium for 7 days. Subsequently, these cells were seeded on porous β-tricalcium phosphate (β-TCP) blocks (10 × 10 × 10 mm^3^, pore size 500 ± 150 μm, Shanghai Bio-lu Biomaterials, Co.) by pipetting cell suspension onto the scaffold. The ADSC/β-TCP constructs were further incubated for an additional 4 h to allow cell attachment before in vivo implantation. These constructs were then subcutaneously transplanted into the dorsal surface of nude mice (8 weeks old) and grown for 6 weeks until animal sacrifice.

Two animal models for heterotopic bone formation were developed to determine the effects of TM-25659 on the osteogenesis and bone formation of ADSCs in vivo. The first model was that ADSCs (2 × 10^6^) were initially induced to osteogenic differentiation by osteogenic medium and TM-25659 for 7 days. Subsequently, these cells were seeded on porous β-TCP blocks (10 × 10 × 10 mm^3^, pore size 500 ± 150 μm, Shanghai Bio-lu Biomaterials, Co.) by pipetting cell suspension onto the scaffold. The ADSC/β-TCP constructs were further incubated for an additional 4 h to allow cell attachment before in vivo implantation. These constructs were then subcutaneously transplanted into the dorsal surface of nude mice (8 weeks old) and grown for 6 weeks until harvest. The other model was that ADSCs (2 × 10^6^) were initially induced to osteogenic differentiation for 7 days and seeded on porous β-TCP blocks as a carrier. After cell attachment on the scaffold, these ADSC/β-TCP constructs were subcutaneously transplanted into nude mice. The animals bearing transplants then received intraperitoneal injection of TM-25659 or vehicle every day for 2 weeks. All samples were harvested 6 weeks after surgical implantation and used for further analyses. All animal experiments were reviewed and approved by the Animal Care and Use Committee of Nanjing Medical University (2016-0258).

### Histological and histomorphometric analyses

All surgical samples from both animal models were fixed in 4% paraformaldehyde upon harvest. These samples were decalcified in 10% ethylenediaminetetraacetic acid (EDTA; pH 7.4) for 4 weeks, followed by dehydration and embedding in paraffin. The sample blocks were sectioned and stained with hematoxylin and eosin (H&E) as well as Masson’s trichrome. Ten fields from each section were randomly selected and captured under the microscope. The newly formed mineralized area was marked using ImageJ software and we calculated the percentage of new bone over the total area. Meanwhile, these sections were also subjected to immunohistochemical staining for OPN and OCN in the newly regenerated bone. Briefly, these decalcified sections were firstly blocked with goat serum and incubated with primary antibodies against OPN (1:200 dilution) and OCN (1:250 dilution) at 4 °C overnight, and further processed with the ABC detection kit (Maixin Biotech). The immunohistochemical staining in each section was visualized under the microscope and recorded. The immunohistochemistry scores for each antigens of interest were semi-quantitatively evaluated on the basis of staining intensity and distribution.

### Statistical analyses

All quantitative data are shown as mean ± SD and compared by Student’s *t* test or one-way analysis of variance (ANOVA) as appropriate. Most experiments were independently repeated two or three times under the same conditions, as indicated. *P* values less than 0.05 (two-sided) were considered statistically significant. All statistical analyses were performed using Graphpad Prism 6.

## Results

### TAZ is upregulated during osteogenic differentiation while it is downregulated during adipogenic differentiation of ADSCs

**Fig. 1:**
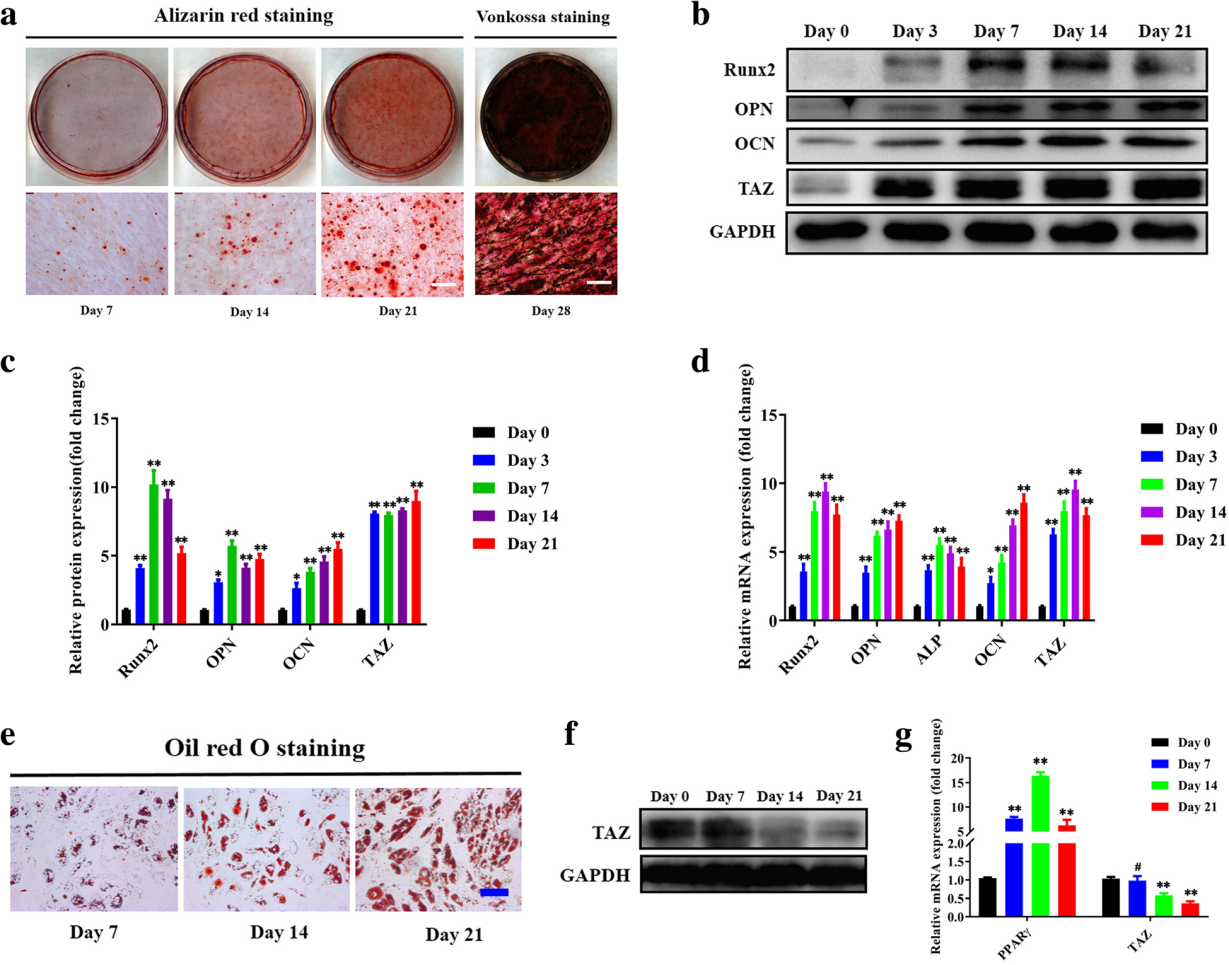
TAZ is upregulated during osteogenesis while it is downregulated during adipogenesis in human ADSCs. **a** Osteogenic differentiation of ADSCs was induced in vitro and monitored by Alizarin Red and von Kossa staining at the indicated time points (days 7, 14, 21, and 28 after induction). Scale bar = 50 μm. **b**,**c** Transcriptional coactivator with PDZ-binding motif (TAZ) protein was significantly increased during the osteogenic differentiation process of ADSCs, concomitant with markedly upregulated expression of the osteogenic markers runt-related transcription factor 2 (Runx2), osteopontin (OPN), and osteocalcin (OCN). Representative images of Western blots from three independent experiments are shown. **d** The mRNA levels of TAZ and the osteogenic markers were significantly increased during ADSC osteogenic differentiation in vitro as measured by quantitative RT-PCR. **e** Adipogenic differentiation of ADSCs was induced in vitro and determined by Oil Red O staining at the indicated time points (days 7, 14, and 21 after induction). Scale bar = 50 μm. **f** TAZ protein was significantly downregulated during the adipogenic differentiation process of ADSCs. Representative images of Western blots from three independent experiments are shown. **g** The mRNA levels of TAZ decreased while the adipogenic marker increased during ADSC adipogenic differentiation in vitro as measured by quantitative RT-PCR. Data shown here are mean ± SD from three independent experiments; ^#^*P* ˃ 0.05, *P < 0.05, **P < 0.01, by ANOVA. ALP alkaline phosphatase, GAPDH glyeraldehyde-3-phosphate dehydrogenase, PPARγ peroxisome proliferator-activated receptor-γ

Accumulating evidence has indicated that TAZ is critically involved in stem cell self-renewal and differentiation in embryonic and MSCs [17, 20, 31]. To further reveal the biological functions of TAZ during osteogenesis in ADSCs, we initially sought to probe the expression pattern of TAZ during human ADSC differentiation. Primary ADSCs from healthy human donors were isolated and expanded in vitro. These cells displayed typical fibroblast- or spindle-like morphology, were positive for CD29 (over 95%), and were negative for CD11b and CD45 (less than 2%) as measured by FACS, and exhibited osteogenic, adipogenic, and chondrogenic differentiation potentials (Fig. 1a and data not shown), thus in part supporting the identity and purity of MSCs from adipose tissue [32, 33]. When these ADSCs were cultured under osteoinductive conditions, increased mineralization was observed over time as evidenced by enhanced Alizarin Red staining and von Kossa staining (Fig. 1a). In parallel, the expression levels of the osteogenic markers Runx2, OPN (osteopontin, encoded by SPP1), and OCN (osteocalcin, encoded by BGLAP) were markedly increased during the osteogenic process. Noticeably, the abundance of TAZ was significantly enhanced from day 3 to day 21 compared with the basal level at day 0 (Fig. 1b, c), while the ratios of its phosphorylated form pTAZ(ser89) over total TAZ protein was significantly reduced during osteogenic differentiation. In addition, the expression of the TAZ paralog YAP as well as the ratios of its phosphorylated protein pYAP(ser127)/YAP were both increased (Additional file 2: Figure S1A, B). Furthermore, when ADSCs were cultured under adipogenic medium, obvious lipid droplet formation was detected at days 7, 14, and 21, with concomitantly increased expression of the adipogenic marker PPARγ over time (Fig. 1e, g). Not unexpectedly, both mRNA and protein levels of TAZ were significantly reduced after adipogenic differentiation (Fig. 1f, g). Furthermore, in agreement with previous studies [17], we also found that TAZ was markedly increased during osteogenic differentiation in MSCs isolated from bone marrow (Additional file 3: Figure S2). Collectively, these data indicate that TAZ is involved in osteogenic and adipogenic differentiation of human ADSCs.

### TAZ depletion impairs osteogenic differentiation of ADSCs in vitro

**Fig. 2:**
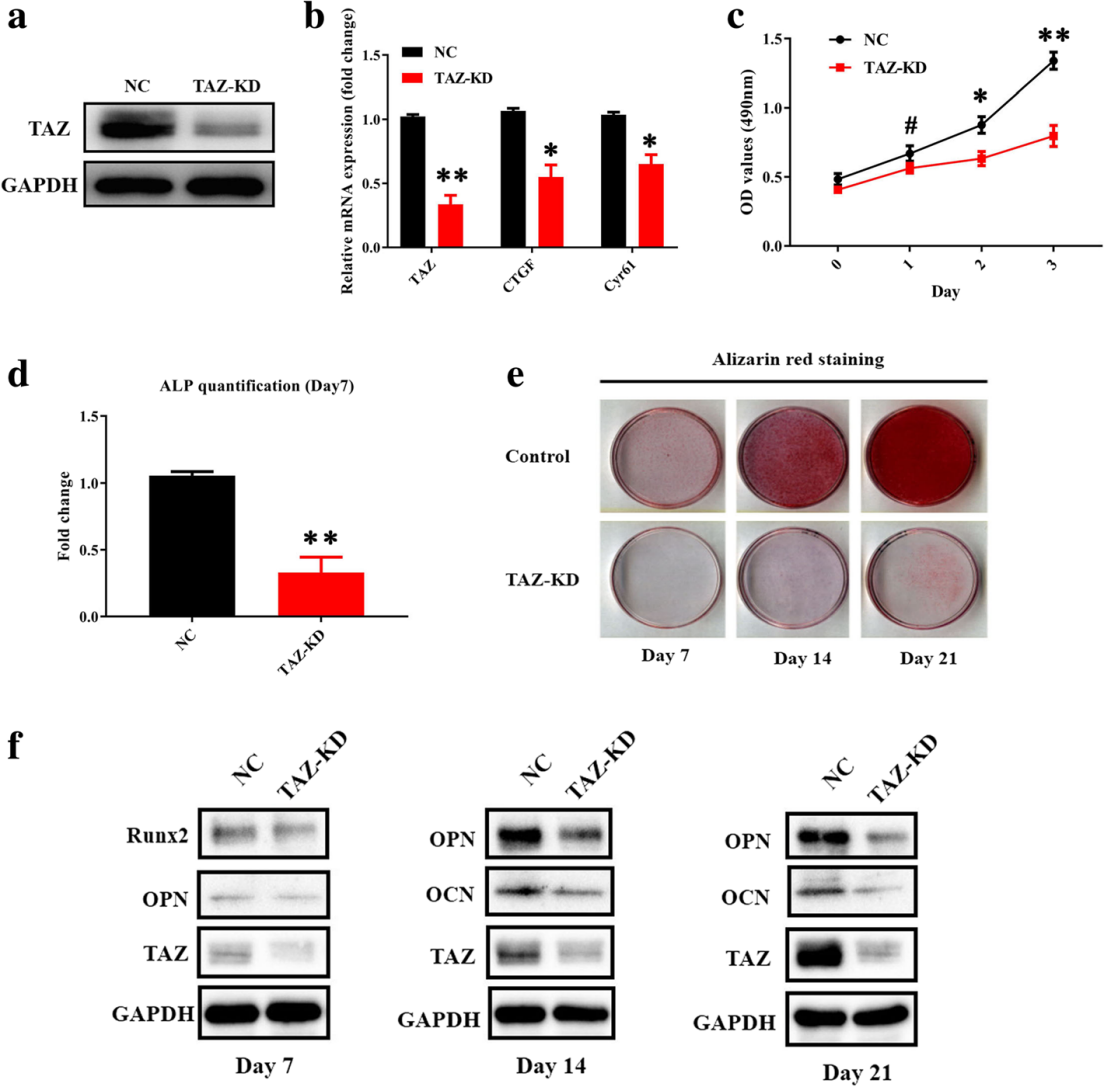
TAZ knockdown impairs osteogenic differentiation of ADSCs in vitro. **a** Transcriptional coactivator with PDZ-binding motif (TAZ) knockdown (KD) in ADSCs infected with shRNA lentiviral particles targeting human TAZ was verified by Western blot. Representative images of Western blots from three independent experiments are shown. **b** TAZ and its downstream targets CTGF and Cyr61 were downregulated in TAZ-knockdown ADSCs as determined by quantitative RT-PCR. **c** Cell proliferation was compromised in TAZ-knockdown ADSCs compared with control cells infected with nontargeting lentiviral vectors with scrambled sequence. **d** Compromised osteogenic differentiation of ADSCs was detected and quantified by alkaline phosphatase (ALP) activity assay following TAZ knockdown of ADSCs induced in vitro at day 7. **e** Reduced mineralization in TAZ-knockdown ADSCs cultured in osteoinductive medium was observed via Alizarin Red staining at days 7, 14, and 21. **f** Significantly reduced expression of TAZ as well as the osteogenic markers osteopontin (OPN) and osteocalcin (OCN) in TAZ-knockdown ADSCs cultured in osteoinductive medium at days 7, 14, and 21 was detected by Western blot. Representative images of Western blots from three independent experiments are shown. Data shown here are mean ± SD from three independent experiments, ^#^*P* ˃ 0.05, *P < 0.05, **P < 0.01, by Student’s *t* test. GAPDH glyeraldehyde-3-phosphate dehydrogenase, NC normal control, OD optical density, Runx2 runt-related transcription factor 2

Given the importance of TAZ underlying MSC differentiation, we next sought to reveal whether TAZ is required for osteogenic differentiation of ADSCs by both loss- and gain-of-function approaches. We initially infected ADSCs with shRNA lentiviral vector targeting human TAZ and obtained the stable cell clones. Depletion of TAZ in ADSCs was verified by marked reduction of TAZ and its well-established downstream targets CTGF and Cyr61 (Fig. 2a, b). The effects of TAZ knockdown on ADSC proliferation and apoptosis were determined by MTT assay and Anexin V-PI double-staining assay. Our data revealed that cell proliferation was significantly impaired in TAZ-depleted cells relative to control cells (Fig. 2c). In addition, the percentage of apoptotic cells in TAZ knockdown and control cells was comparable without a significant difference (data not shown). ADSCs with stable TAZ knockdown were then induced in osteogenic medium to assess the effects of TAZ loss on osteogenesis in vitro. After osteogenic induction, we found that ALP activity was remarkably reduced in TAZ-depleted ADSCs at day 7 and compromised mineralization at days 7, 14, and 21 as assessed by Alizarin Red staining (Fig. 2d, e). Complementary to these findings, the protein abundance of Runx2, OPN, and OCN in TAZ-depleted ADSCs were all significantly lower than those control cells at each time point after osteogenic induction (Fig. 2f). In contrast, TAZ knockdown resulted in enhanced adipogenic differentiation in ADSCs as assessed by Oil Red O staining and enhanced expression of PPARγ at day 14 (Additional file 4: Figure S3).

### Enforced TAZ overexpression promotes osteogenic differentiation of ADSCs in vitro and bone formation in vivo

**Fig. 3:**
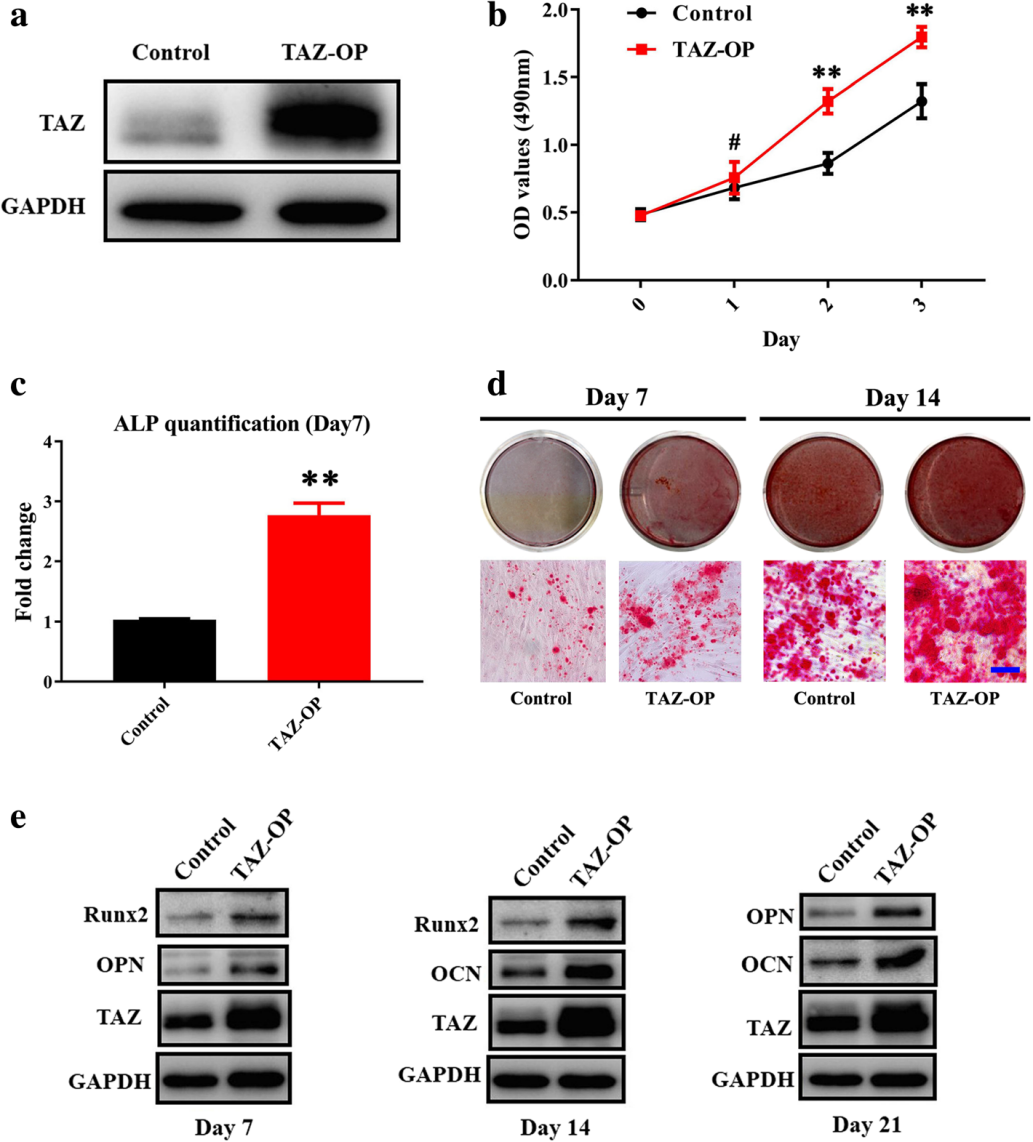
Enforced TAZ overexpression enhances osteogenic differentiation of ADSCs in vitro. **a** Transcriptional coactivator with PDZ-binding motif (TAZ) overexpression (OP) in ADSCs infected with lentiviral particles containing human TAZ cDNA sequence was verified by Western blot. Representative images of Western blots from three independent experiments are shown. **b** Accelerated cell proliferation was detected in TAZ-overexpressing ADSCs compared with control cells infected with empty lentiviral particles via MTT assay. **c** Enhanced osteogenic differentiation of ADSCs was detected and quantified by alkaline phosphatase (ALP) activity assay following TAZ overexpression in ADSCs induced in vitro at day 7. **d** Increased mineralization in TAZ-overexpressing ADSCs cultured in osteoinductive medium was observed via Alizarin Red staining at days 7 and 14. Scale bar = 50 μm. **e** Significantly enhanced expression of TAZ as well as the osteogenic markers osteopontin (OPN) and osteocalcin (OCN) in TAZ-overexpressing ADSCs cultured in osteoinductive medium at days 7, 14, and 21 was detected by Western blot. Representative images of Western blots from three independent experiments are shown. Data shown here are mean ± SD from three independent experiments; ^#^*P* ˃ 0.05, *P < 0.05, **P < 0.01, by Student’s *t* test. GAPDH glyeraldehyde-3-phosphate dehydrogenase, OD optical density, Runx2 runt-related transcription factor 2

To further reinforce the critical roles of TAZ during osteogenic differentiation of ADSCs, we infected ADSCs with lentiviral vectors containing human TAZ cDNA sequence and generated stable cell clones. Enforced TAZ overexpression in ADSCs was verified by Western blot (Fig. 3a). In addition, cell proliferation in ADSCs with TAZ overexpression was significantly higher than control cells infected with empty vector, while no obvious difference in cell apoptosis was observed between these two types of cells (Fig. 3b and data not shown). Subsequently, ADSCs with enforced TAZ overexpression were subject to osteogenic differentiation in vitro. After osteogenic induction, we found that the ALP activity compared with control cells was remarkably higher in TAZ-overexpressing ADSCs at day 7 with enhanced mineralization at day 7 and 14 as assessed by Alizarin Red staining (Fig. 3c, d). Furthermore, the abundance of Runx2, OPN, and OCN protein in TAZ-overexpressing ADSCs was significantly higher than in control cells at each time point following osteogenic induction in vitro (Fig. 3e). Meanwhile, adipogenic potential was compromised in TAZ-overexpressing ADSCs as evidenced by a reduced formation of lip droplets and reduced PPARγ expression (Additional file 4: Figure S3). More importantly, when these ADSCs with stable TAZ overexpression were initiated into osteogenic differentiation and, in turn, loaded onto a porous β-TCP scaffold and subcutaneously transplanted, the H&E and Masson staining indicated much more bone-like structure was formed in TAZ-overexpressing ADSCs compared with control cells without TAZ overexpression (Additional file 5: Figure S4). Taken together, our loss- and gain-of-function studies reveal that TAZ is a critical modular for driving osteogenic differentiation of ADSCs and might be a novel therapeutic target for facilitating bone regeneration and repair.

### TM-25659 promotes osteogenic differentiation of ADSCs in vitro

**Fig. 4:**
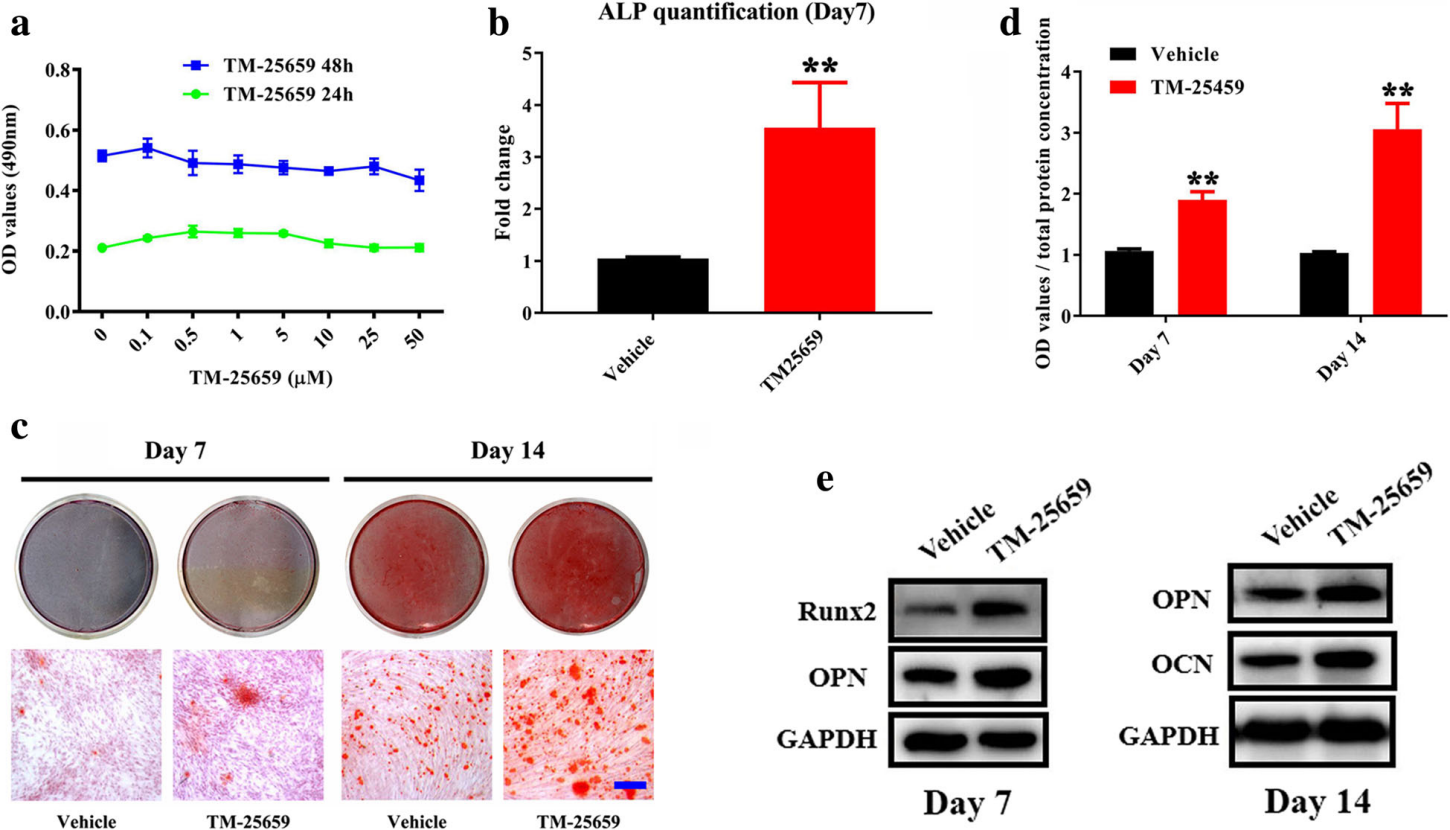
Exposure of TAZ activator TM-25659 promotes osteogenic differentiation of ADSCs in vitro. **a** Cell viability was determined after ADSCs were cultured with diverse concentrations of TM-25659 (0–50 μM) at 24 h and 48 h, respectively. **B** Enhanced osteogenic differentiation of ADSCs was detected and quantified by alkaline phosphatase (ALP) activity assay following ADSC culture with osteoinductive medium and TM-25659 at day 7. **c**, **d** Increased mineralization in ADSCs cultured in osteoinductive medium and TM-25659 was observed and quantified via Alizarin Red staining at days 7 and 14. Scale bar = 50 μm. **e** Significantly increased expression of the osteogenic markers runt-related transcription factor 2 (Runx2), osteopontin (OPN), and osteocalcin (OCN) was observed in ADSCs cultured in osteoinductive medium and TM-25659 at days 7 and 14, respectively. Representative images of Western blots from three independent experiments are shown. Data shown here are mean ± SD from three independent experiments; **P < 0.01, by Student’s *t* test. GAPDH glyeraldehyde-3-phosphate dehydrogenase, OD optical density

Having established the pro-osteogenic roles of TAZ in ADSCs and considering its translational potentials in regenerative medicine, we wondered whether TAZ could be activated by a pharmacological approach and whether such activation could promote osteogenic differentiation and bone regeneration of ADSCs. Recent seminal works have identified TM-25659 as a potent TAZ modulator by high-throughput screening and have demonstrated that it enhanced osteogenic differentiation of C3H10T1/2 cells by facilitating nuclear translocation of TAZ and subsequent Runx2 activation [24, 28]. To test whether TM-25659 affects osteogenic differentiation of ADSCs, we next proceeded to culture ADSCs in osteogenic induction medium supplemented with TM-25659. We firstly determined the effects of TM-25659 on cell viability by culturing ADSCs with diverse concentrations of TM-25659. Our findings revealed that viability of ADSCs was not significantly affected by TM-25659 between 0 and 50 μM at both 24 h and 48 h treatment points. Similar findings were also observed in TM-25659-treated BMSCs (data not shown), therefore suggesting a relatively low cytotoxicity for TM-25659. We found that TM-25659 treatment significantly resulted in increased TAZ protein in the nuclear fraction while it resulted in decreased TAZ in the cytoplasmic compartment in both ADSCs and BMSCs. However, the overall abundance of TAZ was not remarkably affected after TM-25659 exposure (Fig. 4a and Additional file 6: Figure S5). Next, we selected the 10 μM concentration of TM-25659 for the following in vitro experiments largely due to its potent activation of TAZ. After TM-25659 exposure, both proliferation and apoptosis of ADSCs were not markedly changed as assessed by MTT and Anexin V-PI double staining assay (Additional file 7: Figure S6). ADSCs were then induced to osteogenic differentiation in vitro by osteoinductive medium alone or in combination with TM-25659. After osteogenic induction, the ALP activity in ADSCs treated with osteoinductive medium and TM-25659 was significantly higher than in those treated with osteoinductive medium alone at day 7 (Fig. 4b). Moreover, enhanced mineralization at days 7 and 14 was observed in ADSCs treated with osteoinductive medium and TM-25659 as determined by Alizarin Red staining and quantification (Fig. 4c, d). In line with these data, the protein abundance of Runx2, OPN, and OCN was significantly higher in TM-25659-treated ADSCs compared with vehicle-treated cells after osteogenic induction at days 7 and 14, respectively (Fig. 4e). To further verify the pro-osteogenic function of TM-25658 in ADSCs via TAZ, we treated the TAZ-knockdown ADSCs with osteoinductive medium alone or together with TM-25659 for 14 days. As shown in Additional file 8 (Figure S7), we found that the pro-osteogenic effects of TM-25659 were largely attenuated when endogenous TAZ was depleted in ADSCs as evidenced by compromised mineralization and lower mRNA expression of OPN and OCN in TAZ knockdown cells treated with TM-25659 compared with control cells treated with TM-25659. Collectively, these findings revealed that TM-25659 promoted osteogenic differentiation of ADSCs, likely by TAZ.

### TM-25769 promotes TAZ nuclear translocation and interaction between TAZ and Runx2 and enhances OCN transcription in ADSCs

**Fig. 5:**
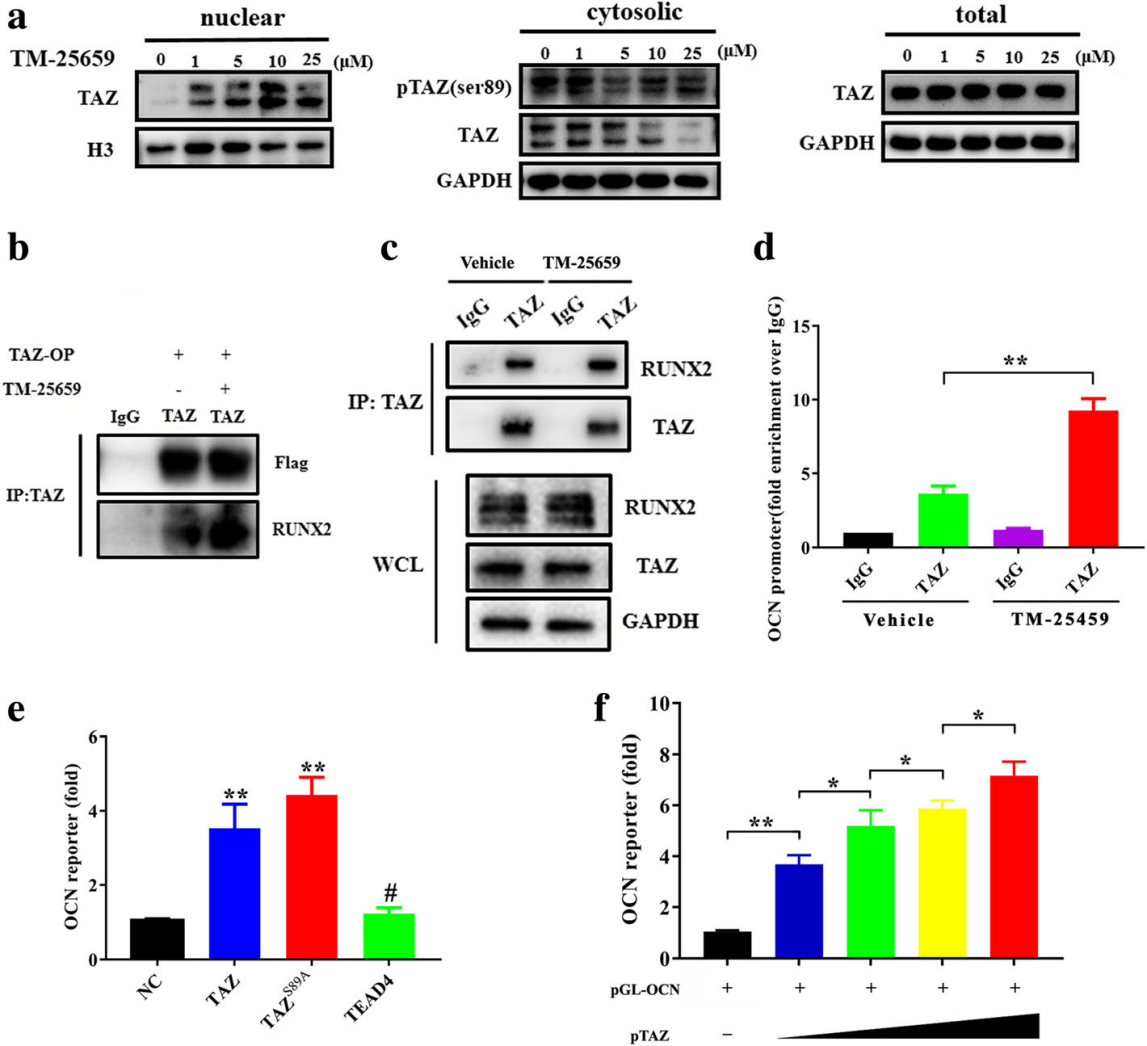
TM-25659 facilitates TAZ nuclear translocation and its binding with Runx2 to potentiate OCN transcription. **a** TM-25659 treatment significantly enhanced transcriptional coactivator with PDZ-binding motif (TAZ) nuclear translocation and decreased its phosphorylation while it did not affect the overall abundance of TAZ in ADSCs as determined by protein nuclear-cytoplasmic fraction and Western blot assay. **b** HEK293T cells were infected with TAZ-overexpressing (OP) lentiviral particles and further treated with TM-25659 (10 μM, 24 h). The cell lysates were immunoprecipitated (IP) with a TAZ antibody or an IgG. Exogenous TAZ protein bound with endogenous Runt-related transcription factor 2 (Runx2) protein was analyzed by Western blot. Representative images from three independent experiments are shown. **c** ADSCs were treated with osteoinductive medium and TM-25659 (10 μM) for 96 h. The cell lysates were then immunoprecipitated with a TAZ antibody or an IgG. Endogenous TAZ protein bound with Runx2 protein was analyzed by Western blot. Representative images from three independent experiments are shown. **d** Occupation of TAZ in the osteocalcin (OCN) promoter was determined by ChIP assay in ADSCs under osteogenic medium and TM-25659 for 96 h. Results are shown as fold-enrichment relative to IgG IP controls. **e** Luciferase activity assay from OCN promoter reporter assay in HEK293T cells cotransfected with indicated constructs and pGL-OCN as well as control phRL-CMV plasmids. **f** Luciferase activity assay from OCN promoter reporter assay in HEK293T cells cotransfected with increased TAZ plasmid together with pGL-OCN, phRL-CMV plasmids. Data shown here are mean ± SD from three independent experiments; ^#^*P* ˃ 0.05, *P < 0.05, **P < 0.01, by ANOVA and Student’s *t* test. GAPDH glyeraldehyde-3-phosphate dehydrogenase, NC normal control

Having revealed the pro-osteogenic functions of TM-265659, presumably via TAZ, in ADSCs we next sought to delineate the molecular mechanisms involved. Previous studies have indicated that TAZ functions as a molecular rheostat for MSC differentiation by coactivating Runx2-dependent OCN transcription while repressing PPARγ-dependent transcription [17, 18]. As shown in Fig. 5a, TM-25659 potently promoted nuclear translocation of TAZ and reduced its phosphorylation (phospho-TAZ (Ser89)), whereas it did not increase the overall abundance of TAZ in ADSCs. To determine whether TM-25659 affects the interaction between TAZ and Runx2, we generated stable TAZ-overexpressing HEK293T cells and treated them with TM-25659. As anticipated, TM-25659 treatment remarkably enhanced the binding of exogenous TAZ protein with endogenous Runx2 protein (Fig. 5b). Furthermore, after ADSCs were cultured with osteoinductive medium and TM-45659 for 7 days, our findings from immunoprecipitation assays revealed that the addition of TM-25659 significantly increased the binding between endogenous TAZ and Runx2 protein (Fig. 5c). Given the fact that TAZ is a Runx2 transcriptional coactivator for OCN expression in C2C12 cells [24], we next wanted to determine whether TM-25659 enhanced OCN transcription by facilitating direct binding of TAZ to the OCN promoter in ADSCs. As shown in Fig. 5d, ADSCs were treated with osteoinductive medium and TM-25659 for 7 days and harvested for ChIP assay. Our results from ChIP assay revealed that, compared with vehicle-treated cells, the occupancy of TAZ in OCN promoter was significantly enhanced by TM-25659. To further substantiate the direct binding between TAZ and the OCN promoter, we engineered the luciferase reporter plasmid containing human OCN promoter (pGL-OCN) and performed luciferase assays. Our data revealed (Fig. 5e, f) that, compared with control, both TAZ wild-type and its constitutively active-type TAZ^S89A^ mutant significantly enhanced the luciferase activities of the OCN promoter construct pGL-OCN. However, TEAD4, one of the primary transcriptional partners of TAZ, failed to induce more luciferase activity of the OCN promoter. This is consistent with recent reports in which TEAD4 was not critically involved in TAZ/Runx2-mediated OCN transcription [34, 35]. Moreover, with the increased abundance of TAZ plasmid used in the reporter assay, the luciferase actives were significantly increased in a dose-dependent manner (Fig. 5f). The change in OCN mRNA in ADSCs following TAZ depletion or enforced overexpression cultured in growth medium was also measured. As indicated in Additional file 9 (Figure S8), TAZ knockdown significantly reduced the mRNA level of OCN, while its overexpression increased OCN transcription in ADSCs. However, other osteogenic markers (Runx2, ALP, and OPN) were not significantly or modestly affected following TAZ manipulation in ADSCs (Additional file 9: Figure S8). Taken together, we believe that TM-25659 treatment facilitates TAZ nuclear translocation which, in turn, forms a protein complex with Runx2. The TAZ-Runx2 complex is then recruited to the promoter of OCN to enhance its transcription and subsequently drive osteogenic differentiation of ADSCs (Additional file 10: Figure S9).

### TM-25659 promotes bone formation of ADSCs in vivo

**Fig. 6:**
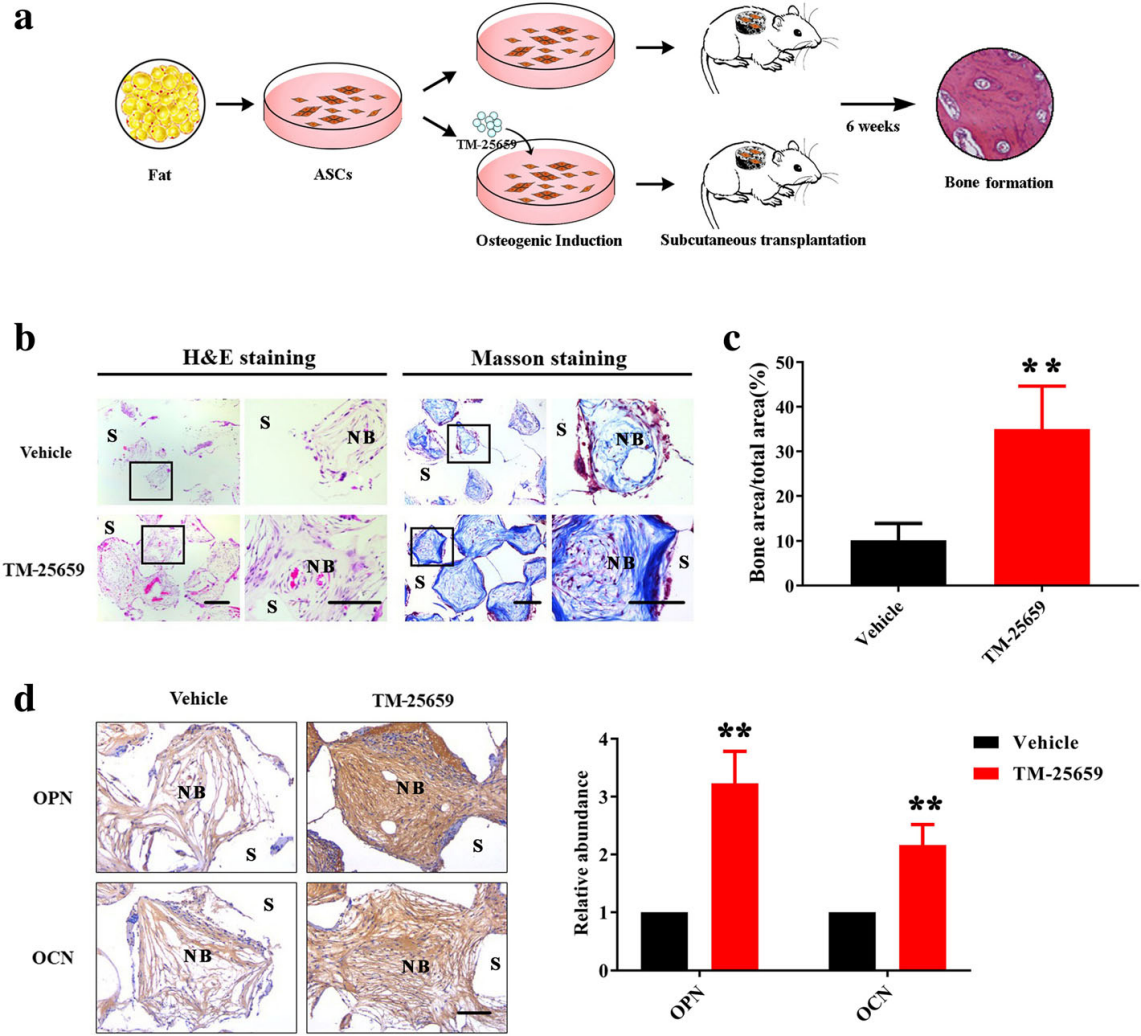
TM-25659 promotes in vivo bone formation of ADSCs. **a** Schematic description of experimental procedure for in vivo transplantation. Adipose-derived stem cells (ASCs) were initially treated with osteoinductive medium and TM-25659 for consecutive 7 days, then harvested, seeded on porous β-TCP blocks as a carrier, and subcutaneously transplanted into nude mice (six animals per experimental group). Six weeks later all transplants were harvested for further analysis. **b** Hematoxylin and eosin (H&E) and Masson trichrome staining revealed markedly enhanced bone formation in samples from ADSCs treated with TM-25659 compared with vehicle-treated samples. Scale bar = 100 μm. **c** Quantification of bone formation in samples indicated significantly more bone formation in ADSCs treated with TM-25659. Ten images of Masson staining (400×) were randomly selected in the slides from two experimental groups and captured under microscopy. The area of new bone in each image was marked using ImageJ and the percentage of new bone over total area was calculated. **d** Immunohistochemical staining of osteopontin (OPN) and osteocalcin (OCN) in samples revealed elevated OPN and OCN abundance in samples from ADSCs treated with TM-25659 compared with vehicle-treated samples. Scale bar = 100 μm. Data are shown as fold-change compared with vehicle-treated samples which were defined as 1.0. Data shown here are mean ± SD from two independent experiments; **P < 0.01, by Student’s *t* test. NB new bone, S scaffold

Having demonstrated the pro-osteogenic roles of TM-25659 in ADSCs in vitro, we then proceeded to determine whether TM-25659 has the capability to enhance bone regeneration in vivo. We firstly developed an in vivo mouse model for ADSC-mediated bone formation. As described in Fig. 6a, ADSCs were initially induced under osteogenic medium alone or supplemented with TM-25659 for 7 days and mixed with porous β-TCP scaffold as a carrier, and then transplanted subcutaneously into the dorsal side of 8-week-old nude mice. This porous β-TCP scaffold has been demonstrated to have favorable biocompatibility, biological safety, and a superior capacity to promote osteogenesis [36, 37]. After 6 weeks, these transplants were harvested and prepared for further analyses. Both H&E and Masson staining showed that ADSCs treated with TM-25659 generated more bone-like structures and collagen deposit than those treated with vehicle (Fig. 6b). Quantitative measurement of bone-like structure areas indicated a remarkable increase in new bone formation in TM-25659-treated samples compared with vehicle-treated samples (Fig. 6c). Furthermore, our data from immunohistochemical staining of OPN and OCN revealed that the abundance of these two markers was significantly higher in TM-25659-treated samples than vehicle-treated samples (Fig. 6d).

**Fig. 7:**
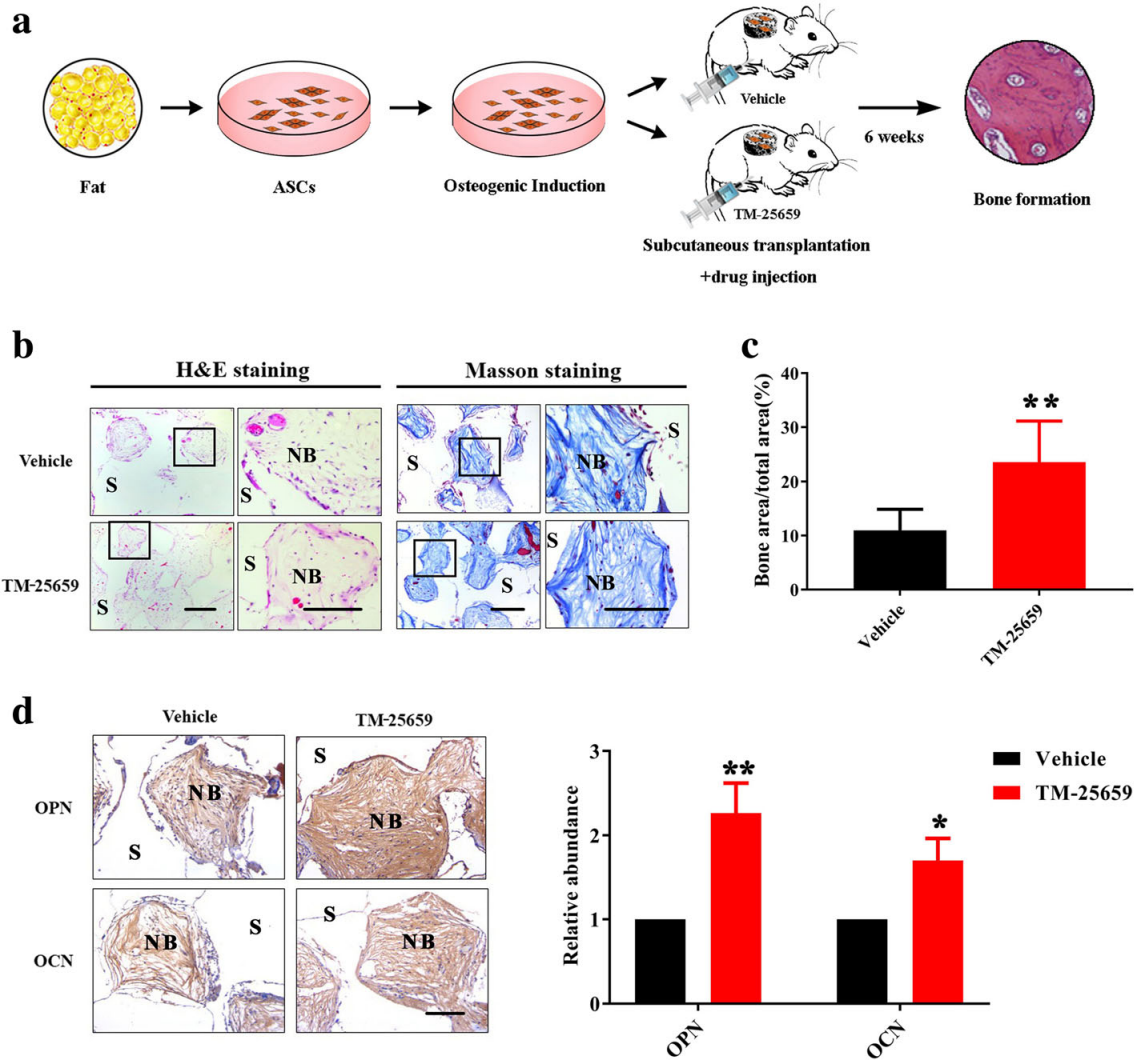
TM-25659 delivery by oral gavage promotes in vivo bone formation of ADSCs. **a** Schematic description of the experimental procedure for in vivo transplantation. Adipose-derived stem cells (ASCs) were initially treated with osteoinductive medium for 7 consecutive days and then harvested, seeded on porous β-TCP blocks as carriers, and subcutaneously transplanted into nude mice (six animals per experimental group). Six weeks later all transplants were harvested for further analysis. **b** Hematoxylin and eosin (H&E) and Masson trichrome staining revealed markedly enhanced bone formation in samples from animals treated with TM-25659 via oral gavage compared with vehicle-treated samples. Scale bar = 100 μm. **c** Quantification of bone formation in samples indicated significantly more bone formation in samples from animals treated with TM-25659. Ten images of Masson staining (400×) were randomly selected in the slides from two experimental groups and captured under microscopy. The area of new bone in each image was marked using ImageJ and the percentage of new bone over total area was calculated. **d** Immunohistochemical staining of osteopontin (OPN) and osteocalcin (OCN) in samples revealed elevated OPN and OCN abundance in samples from animals treated with TM-25659 compared with vehicle-treated samples. Scale bar = 100 μm. Data are shown as fold-change compared with vehicle-treated samples which were defined as 1.0. Data shown here are mean ± SD from two independent experiments; *P < 0.05, **P < 0.01, by Student’s *t* test. NB new bone, S scaffold

Previous reports have documented that TM-25659 has a favorable pharmacokinetic profile after oral and intraperitoneal administration and, in turn, significantly attenuated bone loss in vivo [24, 28, 38]. Considering these advantages, we next sought to further test whether TM-25659 delivery by intraperitoneal administration could promote bone formation in vivo and, if so, would it have significant translational potential. To address this, we developed another animal model for ADSC-mediated bone formation enhanced by intraperitoneal administration of TM-25659. As described in Fig. 7a, ADSCs were first induced under osteogenic conditions for 7 days and then mixed with porous β-TCP scaffold, and transplanted subcutaneously into the dorsal side of nude mice. The animals bearing transplants were randomly divided into two groups: one receiving vehicle and the other receiving TM-25659 by intraperitoneal injection every day for 2 consecutive weeks. After 6 weeks the animals were sacrificed, and all transplants were harvested for further analysis. Both H&E and Masson staining showed that intraperitoneal delivery of TM-25659 significantly promoted bone-like structure formation in vivo as quantified by relative bone area and collagen accumulation (Fig. 7b). Consistently, the abundance of OPN and OCN by immunohistochemical staining was significantly increased in TM-25659-treated samples compared with vehicle-treated samples (Fig. 7c). Taken together, our findings established that TM-25659 promotes osteogenic differentiation and bone formation of ADSCs in vivo.

## Discussion

Mesenchymal stem cells or progenitors residing in adult tissue govern hemostasis and regeneration in response to various damage via self-renewal and multilineage differentiation [39]. These complicated procedures are intricately orchestrated by multiple layers of regulators including transcriptional factors, epigenetic modulators, and noncoding RNAs, among others [8, 9]. Here, our findings reveal that TAZ, the core effector of Hippo signaling, is critically required for osteogenic differentiation of human ADSCs. Pharmacological activation of TAZ by its chemical activator TM-25659 potently promotes osteogenic differentiation and bone formation by ADSCs in vitro and in vivo, likely by recruiting the TAZ-Runx2 complex to the promoter of osteocalcin and, in turn, facilitating its transcription.

### TAZ, a key transcriptional factor involved in stem cell differentiation

Pioneering studies have identified TAZ as a critical molecular rheostat of MSC differentiation by coactivating Runx2-dependent transcription while repressing PPARγ-dependent transcription [17, 18]. A flurry of following studies further established that TAZ is indeed a pivotal mediator during MSC commitment to the osteogenic lineage as well as promoting bone formation in vivo [17, 22]. For example, TAZ was potently activated by Wnt3a or epicatechin gallate via PP1A-mediated dephosphorylation and nuclear translocation to enhance osteogenic differentiation of C3H10T1/2 cells and BMSCs [19, 40, 41]. Noticeably, recent findings have revealed that Snail/Slug regulate bone marrow-derived skeletal stem cell self-renewal and osteogenic differentiation by forming a complex with TAZ or YAP which in turn activates TAZ-YAP/TEAD and Runx2 downstream targets, respectively [20]. Furthermore, the tyrosine kinase ABL and TAZ formed a positive feedback loop by reciprocal stabilization, which was critically required for osteoblast differentiation and embryonic skeletal formation, thus establishing another mechanistically distinct model for MSCs toward the osteoblast lineage [21, 40]. These abovementioned findings have unequivocally demonstrated that TAZ is another master transcriptional factor involved in MSC osteogenic differentiation. In agreement with these previous studies, we revealed in this study that, as with MSCs derived from other tissues, TAZ is required for osteogenic differentiation of human ADSCs as evidenced by increased expression during osteogenic differentiation, decreased mineralization upon TAZ knockdown, and enhanced mineralization following TAZ overexpression. On the other hand, as expected, TAZ inhibited the adipogenic differentiation of ADSCs as revealed by diminished expression during adipogenic differentiation, enhanced lipid droplet formation following TAZ depletion, and vice versa. Here, we cannot rule out the possibility that the TAZ paralog YAP is also implicated in osteogenic/adipogenic differentiation of ADSCs and the precise roles of Hippo signaling upstream of TAZ/YAP underlying these processes remains incompletely known. Collectively, these results all point to the idea that TAZ serves as a potent pro-osteogenic or anti-adipogenic transcriptional factor for ADSCs, which adds another layer of complexity into the regulatory network involving stem cell fate decision, especially toward osteogenic commitment.

### TM-25659, a novel chemical activator of TAZ, promotes osteogenic differentiation of ADSCs

Given the critical roles of TAZ underlying osteogenic differentiation of MSCs from diverse sources, it is of great interest and importance to translate these findings into therapies for bone repair and regeneration. Local delivery of TAZ by lentivirus alleviated the osteoporotic phenotypes in the femoral neck of ovariectomized rats [42]. Several research groups have successfully identified several chemical or bioactive compounds such as TM-25659, epicatechin gallate, Phorbaketal A, IBS008738, kaempferol, and ethacridine as TAZ activators that generated the corresponding biological effects mediated by TAZ in diverse cell types [23, 24, 26, 40, 43, 44]. Of particular interest, TM-25659 robustly enhanced TAZ nuclear localization and increased osteoblast differentiation while it attenuated adipocyte differentiation in C3H10T1/2 and 3 T3-L1 cells [24]. To extend these findings and reinforce the translational potential of TM-25659, we utilized human ADSCs as a cell model to test whether TM-25659 affects their osteogenic differentiation and promotes bone regeneration. Our results indicate that TM-25659 potently facilitates osteogenic differentiation of ADSCs in vitro, as evidenced by increased mineralization and osteogenic marker expression. Moreover, these effects of TM-25659 treatment on ADSCs largely resemble some, although not all (for example, effects on cell proliferation), of the effects of enforced overexpression of TAZ. Therefore, these results support that TM-25659 exerts its functions likely by modulating TAZ in ADSCs.

From the perspective of mechanistic investigations into TM-25659, our data further reveal that TM-25659 treatment potently increases dephosphorylation and nuclear translocation of TAZ, while it does not affect its overall abundance in ADSCs. In agreement with previous studies [17, 24], and as shown in Additional file 10 (Figure S9), our results reveal that TM-25659 facilitates the interaction between TAZ and Runx2 and, in turn, promotes OCN transcription by recruiting the TAZ-Runx2 complex to its promoter region. Together, these findings and previous studies demonstrate that TM-25659 is a chemical activator of TAZ and triggers biological functions mediated by TAZ, irrespective of cell type [24]. Of course, we cannot rule out the possibility that other unidentified mediators beyond TAZ exist that underlie osteogenic differentiation enhanced by TM-25659 [45]. Further studies are warranted to further understand other mediators and mechanisms beyond TAZ underlying TM-25659-mediated MSC differentiation.

### TM-25659 enhances in vivo bone regeneration of ADSCs

Further experimental evidence for the function of TM-25659 derives from our in vivo transplantation studies that revealed that TM-25659 treatment significantly enhances new bone formation when ADSCs cells with porous β-TCP scaffolds were subcutaneously transplanted. The histomorphological examinations of bone formation and immunohistochemical staining of osteogenic markers together collaborated the enhanced bone regeneration in animals inoculated with TM-25659-treated ADSCs. We reasoned that this effect primarily resulted from accelerated osteogenic differentiation of ADSCs induced by TM-25659, rather than its effects on cell proliferation and expansion. Indeed, our data indicate that there are few effects from TM-25659 on cell proliferation and apoptosis of ADSCs in vitro. Therefore, we speculate that cell expansion of ADSCs is not likely to be responsible for enhanced osteogenesis, at least under our experimental conditions.

A line of evidence has revealed that TM-25659 has potent pro-osteogenic and anti-adipogenic activities following in vivo administration by intraperitoneal, intravenous injection, and oral gavage, and also has a favorable pharmacokinetic profile and good bioavailability [24, 28, 38]. To further reinforce the translational potential of TM-25659, we developed another in vivo model in which TM-25659 was given by intraperitoneal injection following subcutaneous transplantation of ADSCs and scaffold. Of note, intraperitoneal delivery of TM-25659 significantly enhanced bone regeneration in vivo. This is consistent with a previous study in which oral administration of TM-25659 prevented bone loss and increased bone mineral density in both ovariectomy-induced and genetic osteoporosis animal models [24]. Notably, all animals tolerated TM-25659 administration well, as evidenced by unaffected activity, weight gains, and normal range of blood tests, etc. This can be associated with the other advantages of TM-25659, such as less cytotoxity and good pharmacokinetic features, as well as good distribution in vivo. Taken together, these findings reveal that in vivo delivery of a TAZ activator such as TM-25659 represents a promising approach to promote bone regeneration and attenuate bone loss, although its dosage and delivery strategy still remains to be optimized.

## Conclusions

In conclusion, our findings confirm the critical roles of TAZ, namely pro-osteogenic and anti-adipogenic, in human ADSCs and further reveals that pharmacological activation of TAZ by its chemical activator TM-25659 promotes osteogenesis and bone regeneration of ADSCs in vitro and in vivo, likely by enhancing TAZ interaction with Runx2 which in turn directly promotes osteocalcin transcription. We believe that therapeutic manipulation of TAZ via a pharmacological approach might represent a novel and promising strategy to facilitate bone repair and regeneration and attenuate aging-related bone loss.

## Additional files

Additional file 1: **Table S1.** Quantitative RT-PCR primer sequences. (DOCX 12 kb)

Additional file 2: **Figure S1.** Expression patterns of TAZ, YAP, and their phosphorylated forms during osteogenic differentiation of ADSCs. (A) Expression of TAZ, YAP, and their phosphorylated proteins during osteogenic differentiation of ADSCs in vitro was examined by Western blot. Representative images of Western blot from three independent experiments are shown and from the same experiment as Fig. 1b. (B) The ratios of phosphorylated TAZ/YAP over total TAZ/YAP at the indicated time points are shown during osteogenic differentiation of ADSCs. (JPEG 903 kb)

Additional file 3: **Figure S2.** TAZ is increased during osteogenic differentiation of BMSCs in vitro. (A) Increased expression of TAZ protein and the osteogenic markers Runx2 and OCN was detected during osteogenic differentiation of human BMSCs at day 7 and 14 by Western blot. Representative images of Western blot from three independent experiments are shown. (B) Increased mRNA levels of TAZ and ALP, Runx2 and OCN were monitored during osteogenic differentiation of BMSCs at day 7 and 14 by quantitative RT-PCR. Data shown here are mean ± SD from three independent experiments; *P < 0.05, **P < 0.01, by Student’s *t* test. (JPEG 181 kb)

Additional file 4: **Figure S3.** TAZ knockdown promotes while its overexpression inhibits adipogenic differentiation of ADSCs in vitro. (A) ADSCs with stable TAZ overexpression (upper panel) or knockdown (lower panel) were cultured in adipogenic inductive medium for 14 days and subjected to Oil Red O staining. Scale bar = 100 μm. (B) The expression of PPARγ mRNA in TAZ knockdown or overexpressing ADSCs cultured in osteogenic induction medium at day 14 was measured by quantitative RT-PCR. Data shown here are mean ± SD from three independent experiments; **P < 0.01, by Student’s *t* test. (JPEG 2608 kb)

Additional file 5: **Figure S4.** Enforced TAZ overexpression in ADSCs promotes bone formation in vivo. (A) H&E and Masson trichrome staining revealed markedly enhanced bone formation in samples from ADSCs with stable TAZ overexpression compared with controls. Scale bar = 100 μm. (B) Quantification of bone formation in samples indicated significantly more bone formation in ADSCs with stable TAZ overexpression. Ten images of Masson trichrome staining (400×) were randomly selected in the slides from two experimental groups and captured under microscopy. The area of new bone in each image was marked using ImageJ software and the percentage of new bone over total area was calculated. Data shown here are mean ± SD from two independent experiments; **P < 0.01, by Student’s *t* test. (JPEG 470 kb)

Additional file 6: **Figure S5.** TM-25659 exposure promotes TAZ nuclear translocation and decreases its phosphorylation, but merely affects its total abundance in BMSCs. BMSCs were cultured in proliferative medium and TM-25659 (10 μM) for 72 h and harvested for nuclear cytoplasmic fraction and Western blot assay. Representative images of Western blots from three independent experiments are shown. (JPEG 117 kb)

Additional file 7: **Figure S6.** TM-25659 treatment does not affect cell proliferation and apoptosis in ADSCs in vitro. (A) Cell proliferation was not significantly affected by TM-25659 treatment (10 μM) as determined by MTT assay. (B) Cell apoptosis was not significantly affected by TM-25659 treatment (10 μM, 48 h) as measured by Annexin V-FITC assay. Data shown here are mean ± SD from two independent experiments; ^#^*P* ˃ 0.05, by Student’s *t* test. (JPEG 600 kb)

Additional file 8: **Figure S7.** The pro-osteogenic roles of TM-25659 were largely abrogated in TAZ-knockdown ADSCs. (A) Quantification data of Alizarin Red staining in TAZ-knockdown ADSCs which were cultured in osteoinductive medium in the presence or absence of TM-25659 at day 7. (B) Expression of OPN and OCN mRNA in TAZ-knockdown ADSCs which were cultured in osteoinductive medium in the presence or absence of TM-25659 at day 7 as assessed by quantitative RT-PCR. Data shown here are mean ± SD from three independent experiments; ^#^*P* ˃ 0.05, *P < 0.05, **P < 0.01, by Student’s *t* test. (JPEG 418 kb)

Additional file 9: **Figure S8.** TAZ knockdown significantly decreases while its overexpression increases the expression of OCN mRNA in ADSCs cultured in growth medium. The abundance of OCN, Runx2, ALP, and OPN mRNA was assessed in stable TAZ-knockdown (A) or overexpressing ADSCs (B) via quantitative RT-PCR. Data shown here are mean ± SD from three independent experiments; ^#^*P* ˃ 0.05, *P < 0.05, **P < 0.01, by Student’s *t* test. (JPEG 697 kb)

Additional file 10: **Figure S9.** A model depicting the proposed mechanisms for TAZ activated by TM-25659 to facilitate the osteogenic differentiation of ADSCs. (JPEG 4161 kb)

## Abbreviations

ABL1: Abelson murine leukemia via oncogene 1
ADSC: Adipose-derived stem cell
ALP: Alkaline phosphatase
ANOVA: Analysis of variance
BMSC: Bone mesenchymal stem cell
ChIP: Chromatin immunoprecipitation
DMEM: Dulbecco’s modified Eagle’s medium
FACS: Fluorescence-activated cell sorting
FBS: Fetal bovine serum
GAPDH: Glyceraldehyde-3-phosphate dehydrogenase
H&E: Hematoxylin and eosin
MSC: Mesenchymal stem cell
OCN: Osteocalcin
OPN: Osteopontin
PCR: Polymerase chain reaction
PPARγ: Peroxisome proliferator-activated receptor-γ
Runx2: Runt-related transcription factor 2
sh: Short hairpin
TAZ: Transcriptional coactivator with PDZ-binding motif
TCP: Tricalcium phosphate
TEAD: TEA-ATTS DNA-binding domain
TGF: Transforming growth factor
