# Supplementary material for: Pharmacological activation of TAZ enhances osteogenic differentiation and bone formation of adipose-derived stem cells
Source: Stem Cell Res Ther. 2018 Mar 7;9:53. doi: 10.1186/s13287-018-0799-z (PMC5842656; doi:10.1186/s13287-018-0799-z)
Supplement: Supplementary file 1 — Table S1. Quantitative RT-PCR primer sequences. (DOCX 12 kb) [file 13287_2018_799_MOESM1_ESM.docx]

**Additional file 1: Table S1 Quantitative RT-PCR primer sequences**

| **Target (human)** | **Forward** | **Reverse** |
| --- | --- | --- |
| **TAZ** | GTTTATGGGACAGTCCGGGAG | AGTCTAAGGGCTTCGGCTCT |
| **CTGF** | GCCACAAGCTGTCCAGTCTAATCG | TGCATTCTCCAGCCATCAAGAGAC |
| **Cyr61** | ATGAATTGATTGCAGTTGGAAA | TAAAGGGTTGTATAGGATGCGA |
| **ALP** | TCATGTTCCTGGGAGATGGTATG | GCATTAGCTGATAGGCGATGTCC |
| **Runx2** | TCGCCAGGCTTCATAGCAAA | GGCCTTGGGTAAGGCAGATT |
| **OPN (encoded by SPP1)** | CTTCTCAGCCAAACGCCGA | CATCTGTTGTGGAGGGGTAGG |
| **OCN (encoded by BGLAP)** | TCCTTTGGGGTTTGGCCTAC | CCAGCCTCCAGCACTGTTTA |
| **PPARγ** | AGCAAACCCCTATTCCATGCT | CACGGAGCTGATCCCAAAGT |
| **GAPDH** | AGGTGAAGGTCGGAGTCAAC | AGTTGAGGTCAATGAAGGGG |
