# Supplementary figures and images for: Pharmacological activation of TAZ enhances osteogenic differentiation and bone formation of adipose-derived stem cells
Source: Stem Cell Res Ther. 2018 Mar 7;9:53. doi: 10.1186/s13287-018-0799-z (PMC5842656; doi:10.1186/s13287-018-0799-z)

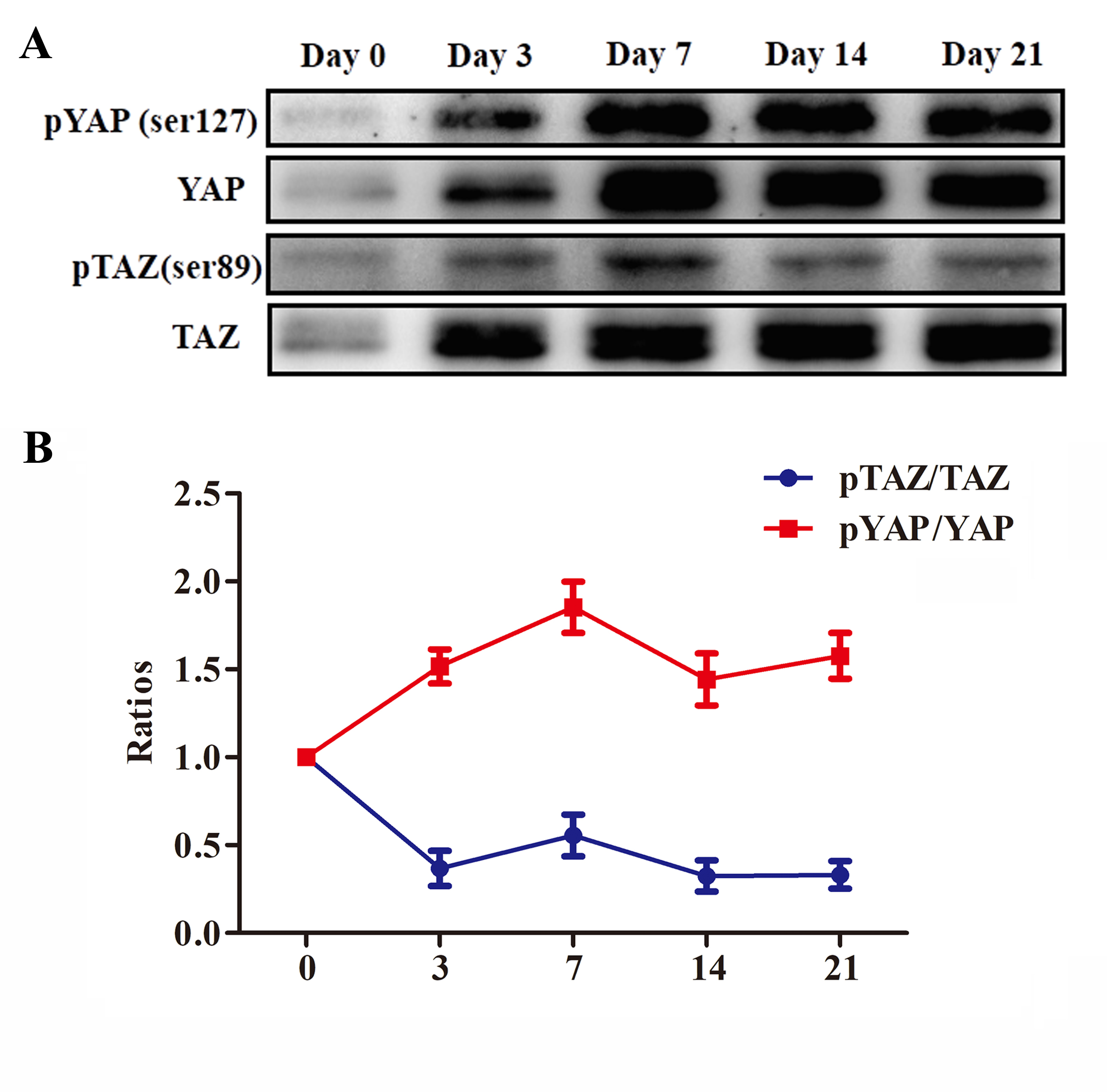

Supplement: Supplementary file 2 — Figure S1. Expression patterns of TAZ, YAP, and their phosphorylated forms during osteogenic differentiation of ADSCs. (A) Expression of TAZ, YAP, and their phosphorylated proteins during osteogenic differentiation of ADSCs in vitro was examined by Western blot. Representative images of Western blot from three independent experiments are shown and from the same experiment as Fig. 1b. (B) The ratios of phosphorylated TAZ/YAP over total TAZ/YAP at the indicated time points are shown during osteogenic differentiation of ADSCs. (JPEG 903 kb) [file 13287_2018_799_MOESM2_ESM.jpg]

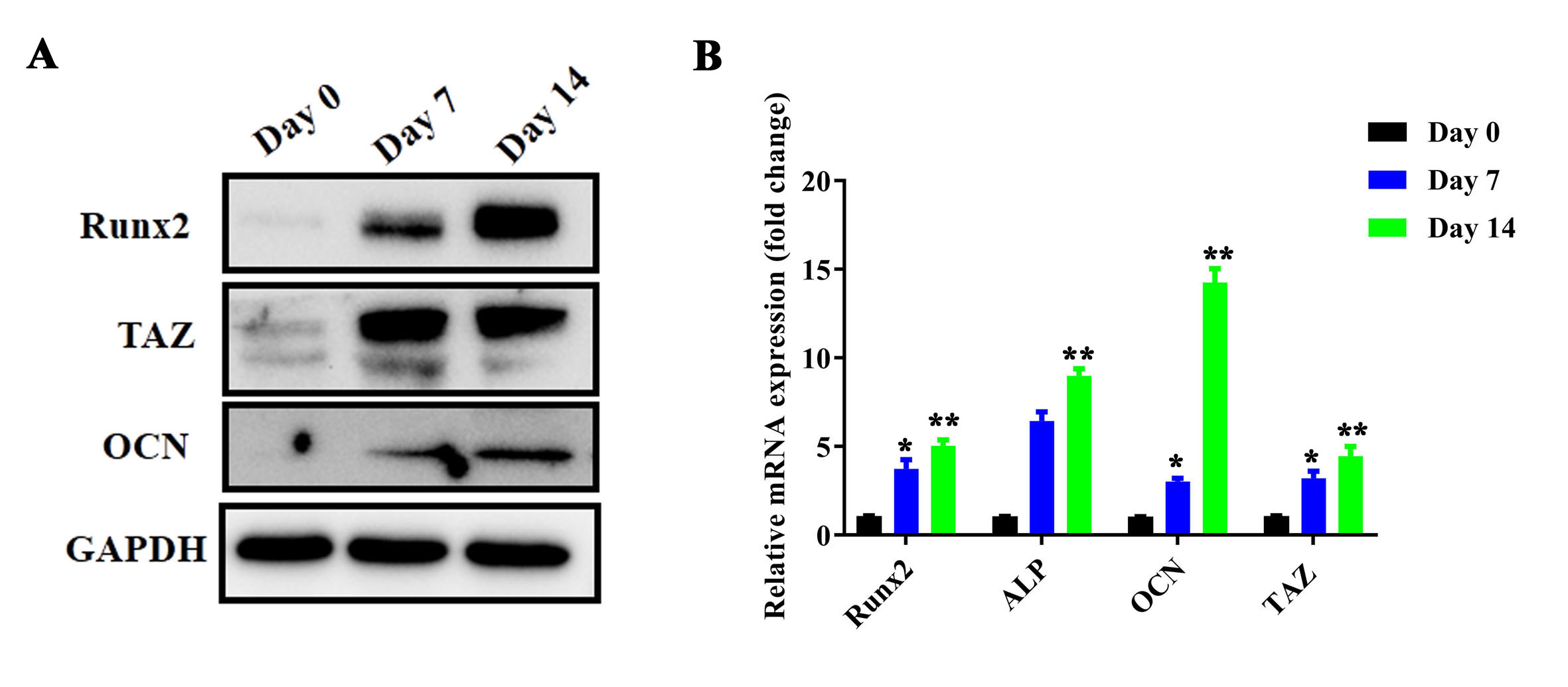

Supplement: Supplementary file 3 — Figure S2. TAZ is increased during osteogenic differentiation of BMSCs in vitro. (A) Increased expression of TAZ protein and the osteogenic markers Runx2 and OCN was detected during osteogenic differentiation of human BMSCs at day 7 and 14 by Western blot. Representative images of Western blot from three independent experiments are shown. (B) Increased mRNA levels of TAZ and ALP, Runx2 and OCN were monitored during osteogenic differentiation of BMSCs at day 7 and 14 by quantitative RT-PCR. Data shown here are mean ± SD from three independent experiments; *P < 0.05, **P < 0.01, by Student’s t test. (JPEG 181 kb) [file 13287_2018_799_MOESM3_ESM.jpg]

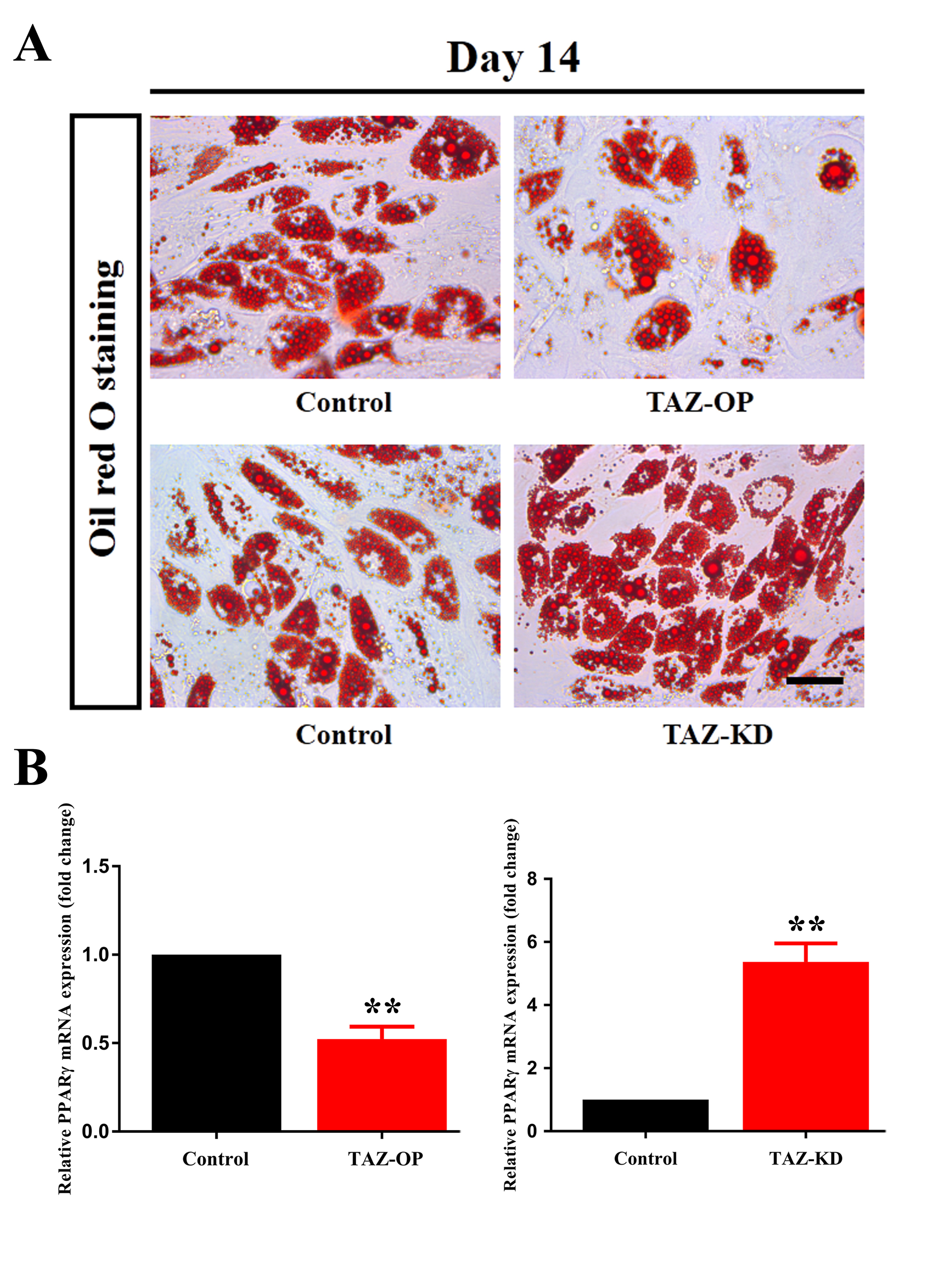

Supplement: Supplementary file 4 — Figure S3. TAZ knockdown promotes while its overexpression inhibits adipogenic differentiation of ADSCs in vitro. (A) ADSCs with stable TAZ overexpression (upper panel) or knockdown (lower panel) were cultured in adipogenic inductive medium for 14 days and subjected to Oil Red O staining. Scale bar = 100 μm. (B) The expression of PPARγ mRNA in TAZ knockdown or overexpressing ADSCs cultured in osteogenic induction medium at day 14 was measured by quantitative RT-PCR. Data shown here are mean ± SD from three independent experiments; **P < 0.01, by Student’s t test. (JPEG 2608 kb) [file 13287_2018_799_MOESM4_ESM.jpg]

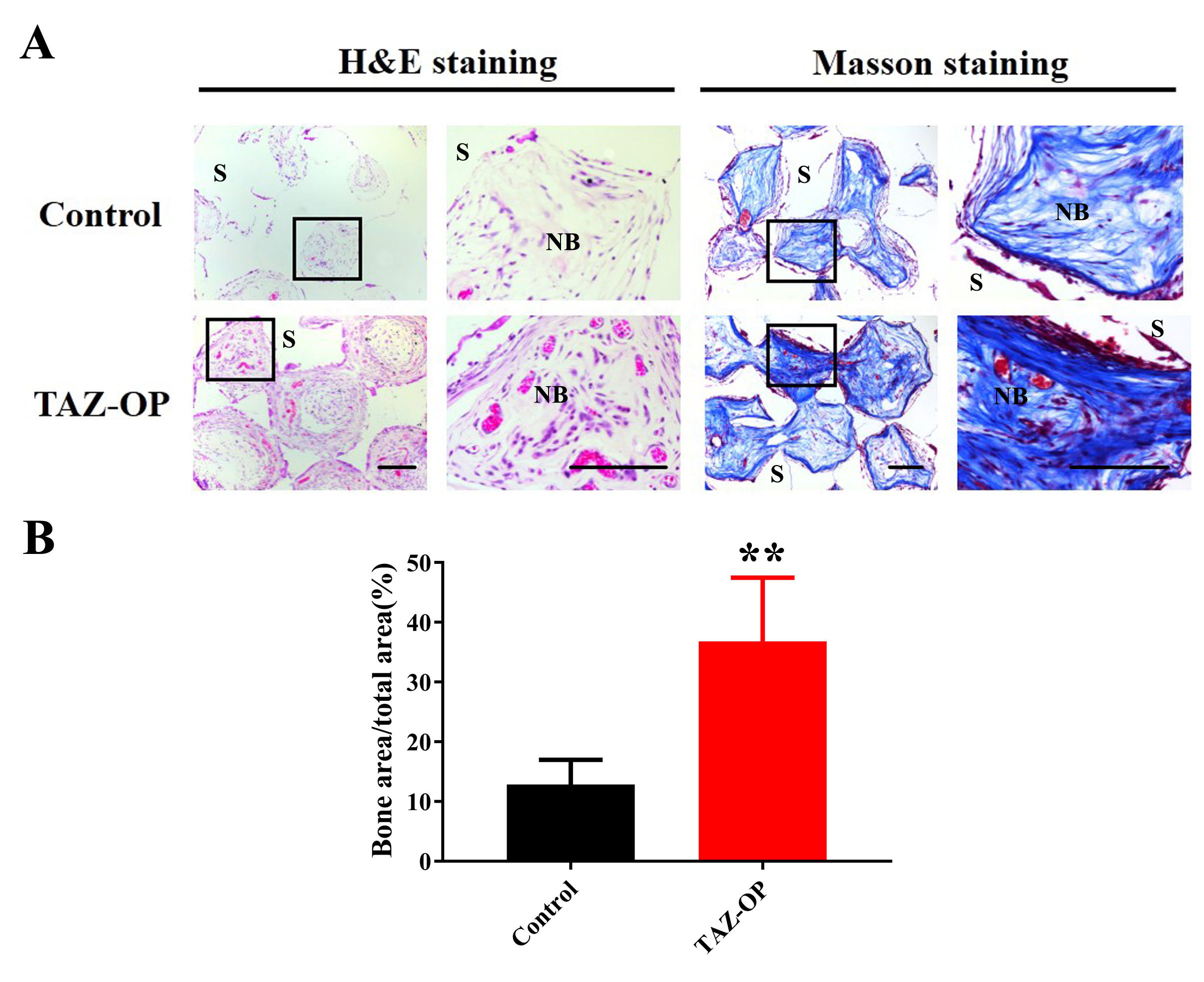

Supplement: Supplementary file 5 — Figure S4. Enforced TAZ overexpression in ADSCs promotes bone formation in vivo. (A) H&E and Masson trichrome staining revealed markedly enhanced bone formation in samples from ADSCs with stable TAZ overexpression compared with controls. Scale bar = 100 μm. (B) Quantification of bone formation in samples indicated significantly more bone formation in ADSCs with stable TAZ overexpression. Ten images of Masson trichrome staining (400×) were randomly selected in the slides from two experimental groups and captured under microscopy. The area of new bone in each image was marked using ImageJ software and the percentage of new bone over total area was calculated. Data shown here are mean ± SD from two independent experiments; **P < 0.01, by Student’s t test. (JPEG 470 kb) [file 13287_2018_799_MOESM5_ESM.jpg]

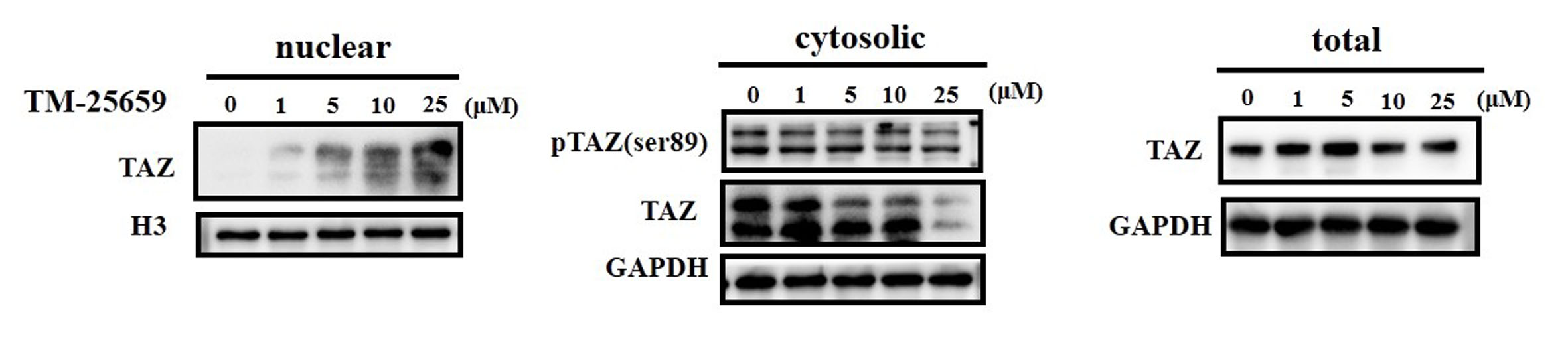

Supplement: Supplementary file 6 — Figure S5. TM-25659 exposure promotes TAZ nuclear translocation and decreases its phosphorylation, but merely affects its total abundance in BMSCs. BMSCs were cultured in proliferative medium and TM-25659 (10 μM) for 72 h and harvested for nuclear cytoplasmic fraction and Western blot assay. Representative images of Western blots from three independent experiments are shown. (JPEG 117 kb) [file 13287_2018_799_MOESM6_ESM.jpg]

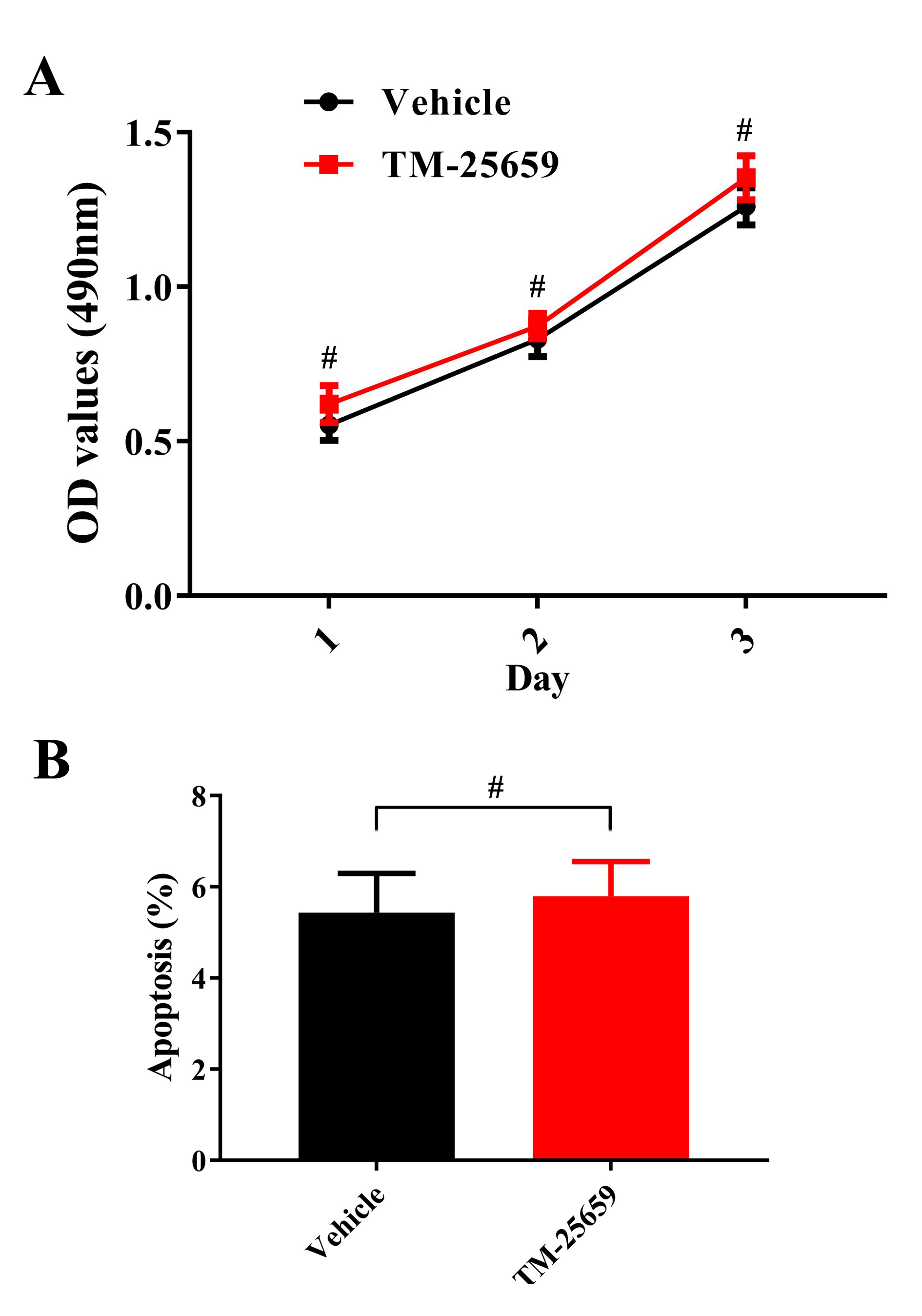

Supplement: Supplementary file 7 — Figure S6. TM-25659 treatment does not affect cell proliferation and apoptosis in ADSCs in vitro. (A) Cell proliferation was not significantly affected by TM-25659 treatment (10 μM) as determined by MTT assay. (B) Cell apoptosis was not significantly affected by TM-25659 treatment (10 μM, 48 h) as measured by Annexin V-FITC assay. Data shown here are mean ± SD from two independent experiments; #P ˃ 0.05, by Student’s t test. (JPEG 600 kb) [file 13287_2018_799_MOESM7_ESM.jpg]

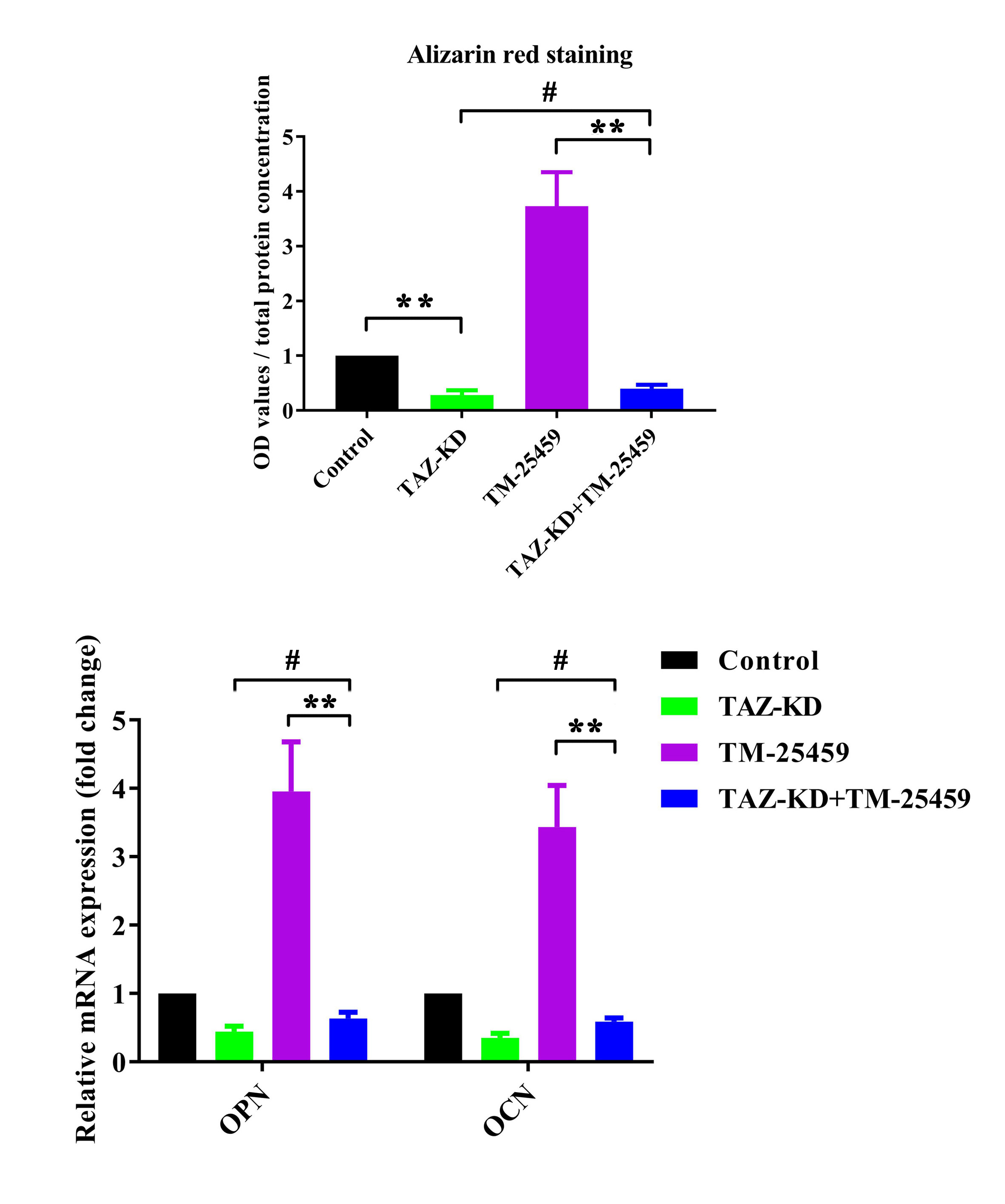

Supplement: Supplementary file 8 — Figure S7. The pro-osteogenic roles of TM-25659 were largely abrogated in TAZ-knockdown ADSCs. (A) Quantification data of Alizarin Red staining in TAZ-knockdown ADSCs which were cultured in osteoinductive medium in the presence or absence of TM-25659 at day 7. (B) Expression of OPN and OCN mRNA in TAZ-knockdown ADSCs which were cultured in osteoinductive medium in the presence or absence of TM-25659 at day 7 as assessed by quantitative RT-PCR. Data shown here are mean ± SD from three independent experiments; #P ˃ 0.05, *P < 0.05, **P < 0.01, by Student’s t test. (JPEG 418 kb) [file 13287_2018_799_MOESM8_ESM.jpg]

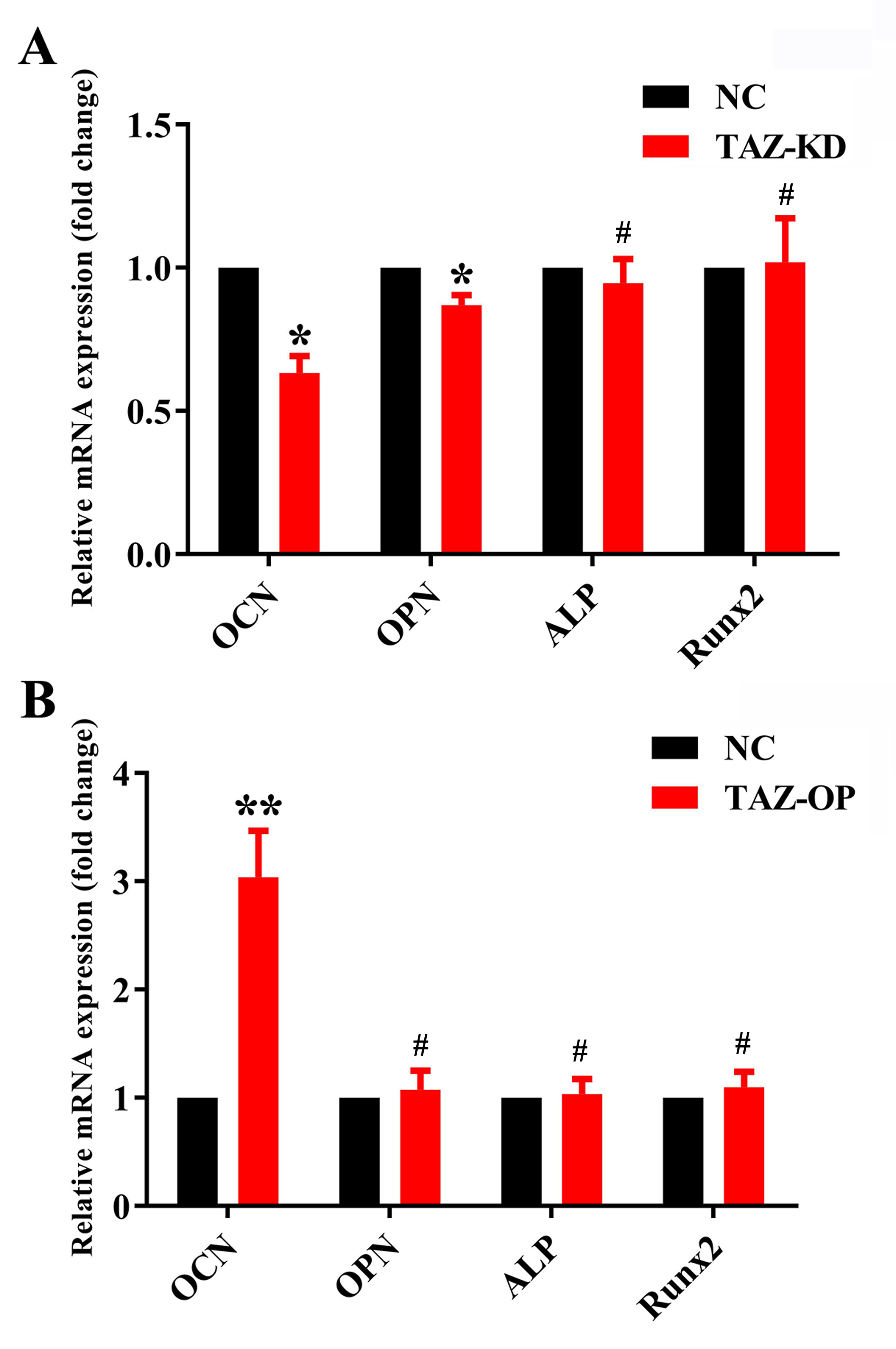

Supplement: Supplementary file 9 — Figure S8. TAZ knockdown significantly decreases while its overexpression increases the expression of OCN mRNA in ADSCs cultured in growth medium. The abundance of OCN, Runx2, ALP, and OPN mRNA was assessed in stable TAZ-knockdown (A) or overexpressing ADSCs (B) via quantitative RT-PCR. Data shown here are mean ± SD from three independent experiments; #P ˃ 0.05, *P < 0.05, **P < 0.01, by Student’s t test. (JPEG 697 kb) [file 13287_2018_799_MOESM9_ESM.jpg]

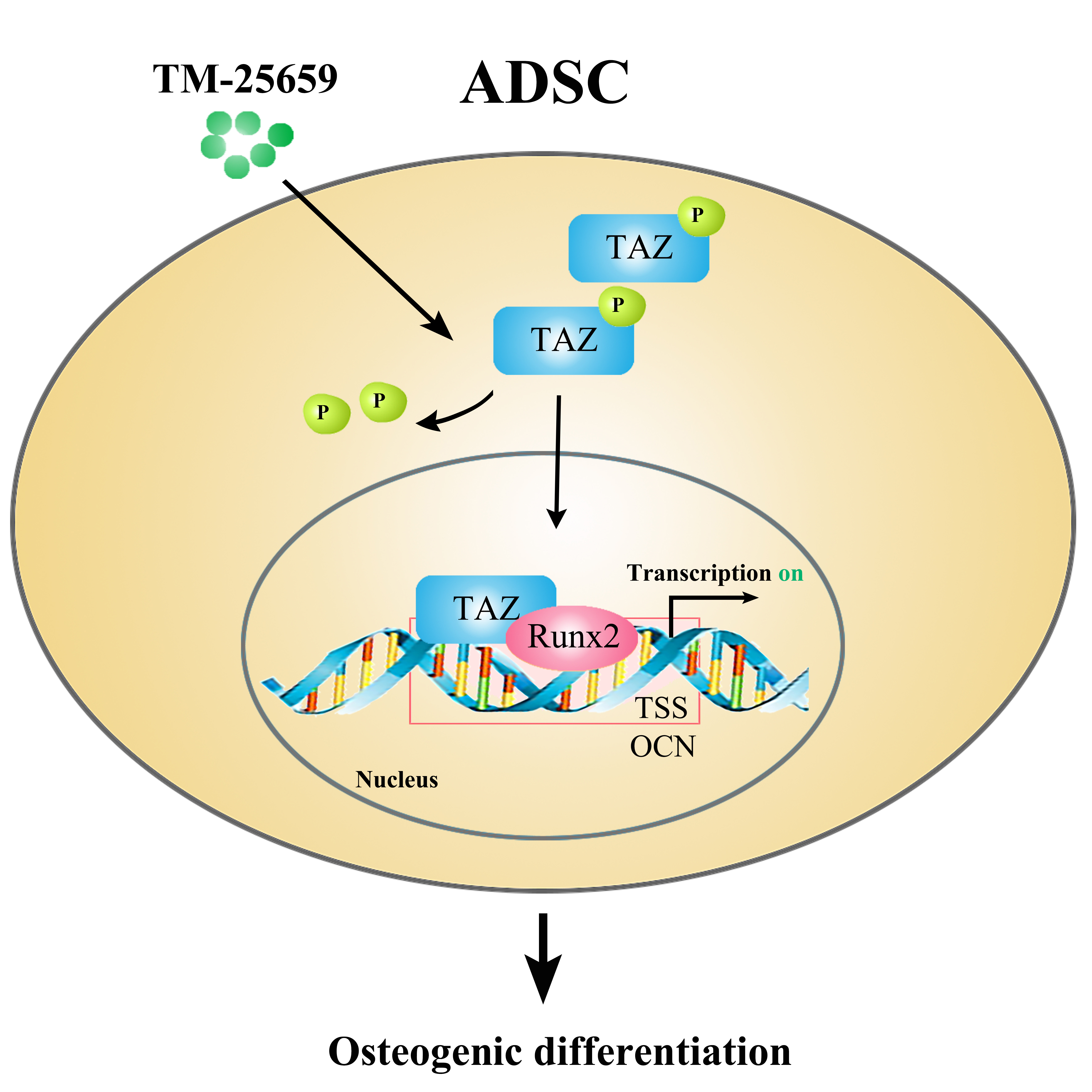

Supplement: Supplementary file 10 — Figure S9. A model depicting the proposed mechanisms for TAZ activated by TM-25659 to facilitate the osteogenic differentiation of ADSCs. (JPEG 4161 kb) [file 13287_2018_799_MOESM10_ESM.jpg]
